# Cost and quality of operational larviciding using drones and smartphone technology

**DOI:** 10.1186/s12936-023-04713-0

**Published:** 2023-09-27

**Authors:** Andy Hardy, Khamis Haji, Faiza Abbas, Juma Hassan, Abdullah Ali, Yussuf Yussuf, Jackie Cook, Laura Rosu, Arnon Houri-Yafin, Arbel Vigodny, Gregory Oakes, Silas Majambere, Eve Worrall

**Affiliations:** 1 Deptartment of Geography and Earth Sciences, Aberystwyth University, Aberystwyth, UK; 2 Zanzibar Malaria Elimination Programme, Zanzibar, Tanzania; 3 PharmAccess Foundation, Dar Es Salaam, Tanzania; 4 Ministry of Health, Revolutionary Government of Zanzibar, Zanzibar, Tanzania; 5 Tanzanian Flying Labs, Dar Es Salaam, Tanzania; 6 MRC International Statistics and Epidemiology Group, London School Hygiene and Tropical Medicine, London, UK; 7 Liverpool School of Tropical Medicine, London, UK; 8 Zzapp Malaria, Jerusalem, Israel; 9 Valent BioSciences, Oslo, Norway

## Abstract

**Background:**

Larval Source Management (LSM) is an important tool for malaria vector control and is recommended by WHO as a supplementary vector control measure. LSM has contributed in many successful attempts to eliminate the disease across the Globe. However, this approach is typically labour-intensive, largely due to the difficulties in locating and mapping potential malarial mosquito breeding sites. Previous studies have demonstrated the potential for drone imaging technology to map malaria vector breeding sites. However, key questions remain unanswered related to the use and cost of this technology within operational vector control.

**Methods:**

Using Zanzibar (United Republic of Tanzania) as a demonstration site, a protocol was collaboratively designed that employs drones and smartphones for supporting operational LSM, termed the Spatial Intelligence System (SIS). SIS was evaluated over a four-month LSM programme by comparing key mapping accuracy indicators and relative costs (both mapping costs and intervention costs) against conventional ground-based methods. Additionally, malaria case incidence was compared between the SIS and conventional study areas, including an estimation of the incremental cost-effectiveness of switching from conventional to SIS larviciding.

**Results:**

The results demonstrate that the SIS approach is significantly more accurate than a conventional approach for mapping potential breeding sites: mean % correct per site: SIS = 60% (95% CI 32–88%, p = 0.02), conventional = 18% (95% CI − 3–39%). Whilst SIS cost more in the start-up phase, overall annualized costs were similar to the conventional approach, with a simulated cost per person protected per year of $3.69 ($0.32 to $15.12) for conventional and $3.94 ($0.342 to $16.27) for SIS larviciding. The main economic benefits were reduced labour costs associated with SIS in the pre-intervention baseline mapping of habitats. There was no difference in malaria case incidence between the three arms. Cost effectiveness analysis showed that SIS is likely to provide similar health benefits at similar costs compared to the conventional arm.

**Conclusions:**

The use of drones and smartphones provides an improved means of mapping breeding sites for use in operational LSM. Furthermore, deploying this technology does not appear to be more costly than a conventional ground-based approach and, as such, may represent an important tool for Malaria Control Programmes that plan to implement LSM.

**Supplementary Information:**

The online version contains supplementary material available at 10.1186/s12936-023-04713-0.

## Background

Malaria transmission has substantially reduced over the previous few decades, with much of the success due to interventions targeting the mosquito vectors, such as bed nets and indoor residual spraying (IRS) [1]. However, malaria remains persistent across much of sub-Saharan Africa [2, 3] and insecticide resistance in malaria vectors [4, 5], low population coverage of interventions [6, 7], outdoor vector biting [8–11] and transmission led by secondary vector species [12] are key factors undermining the effectiveness of the most successful malaria control tools.

Larval Source Management (LSM) aims to reduce malaria vector densities by treating or managing water sources where mosquitoes lay eggs and larvae develop. Its use has led to significant reductions in malaria transmission across a variety of environments [7, 13, 14] and represented a core strategy behind the elimination of malaria in Brazil, Italy, USA, Israel, Sri Lanka and China [15–18]. Importantly, LSM tackles outdoor biting mosquitoes [19, 20] and has a community impact, benefitting the population regardless of their economic status and structure of housing [8, 21]. Despite its historical success, logistical challenges and resource constraints [3, 22], particularly where mosquito larval habitats are extensive or difficult to locate, have led the World Health Organization (WHO) to recommend larviciding only where habitats are “few, fixed and findable”.

Whilst novel larvicide products and approaches for its delivery have been developed [23–25], an obvious area for improvement relates to breeding site mapping: timely and precise spatial intelligence regarding the location of potential breeding sites could revolutionize the way in which LSM is carried out. Recent years have seen a rapid development in mapping technologies with the potential to transform how malarial mosquito breeding sites are located and treated.

The rise in availability of sophisticated, user-friendly and relatively low-cost drones, along with other digital tools, such as smartphones, has resulted in a number of studies testing their use for mapping malaria mosquito larval habitats across different landscapes [26–31]. Yet, key questions need to be answered before drones are added to the vector control toolbox: Can drone and smartphone technology be integrated into an operational LSM programme? Do drones provide a more accurate mapping method compared to conventional ground-based approaches? Can drone-mapped information be easily delivered to larviciding operatives? Do drones represent a worthwhile investment for NMCPs?

In collaboration with the Zanzibar Malaria Elimination Programme (ZAMEP) a study was designed to answer these questions and to evaluate the use of drone and smartphone technology within the context of operational larviciding. The aim of this study was to assess the accuracy, costs and cost-effectiveness of a drone and smartphone-based mapping system (termed the Spatial Intelligence System: SIS) compared to conventional larviciding and to standard of care.

## Methods

### Study location

**Fig. 1:**
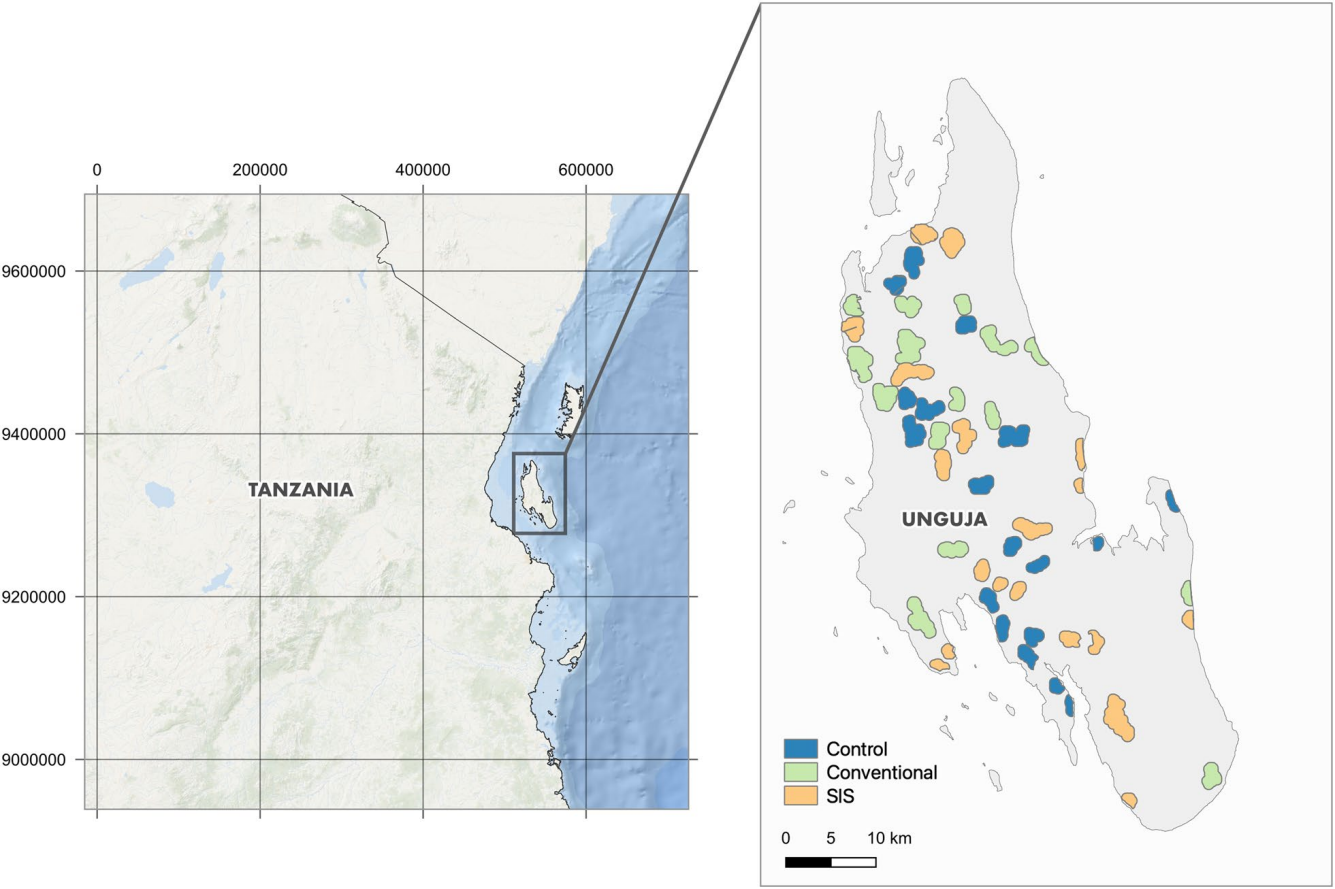
Location of the study cluster locations in Unguja, Zanzibar Archipelago, United Republic of Tanzania

Zanzibar, an island nation part of the Republic of Tanzania (Fig. 1), is a malaria pre-elimination setting, with low residual malaria transmission (community parasite prevalence below 1%) [32]. Historically, transmission is seasonal with a peak in cases following the long rains which take place from March to May [32]. The major malaria vector in Zanzibar is *Anopheles arabiensis* accounting for > 98% of adult mosquitoes caught indoors and outdoors [33].

Current malaria interventions in Zanzibar include periodic mass distribution of LLINs (every three years), effective diagnosis and treatment of clinical malaria, and a comprehensive case surveillance system (the Malaria Case Notification system or MCN) involving passive detection at health facilities, and a detailed follow up at the household level to determine whether the case was likely locally acquired or imported. Additionally, annual targeted IRS takes place in transmission hotspots (determined using the surveillance system) before the rainy season. LSM (using larviciding) has been included in Zanzibar’s strategic plan for malaria elimination, but it has yet to be routinely implemented, mainly due to the challenges in locating water bodies as well as inadequate financing.

### Study design

This study addresses four objectives: (1) Mapping accuracy: assess the accuracy of SIS compared to conventional mapping in terms of their ability to identify (i) water bodies and (ii) specifically identify *Anopheles* breeding sites. (2) Cost analysis: assess and compare the costs of SIS versus conventional larviciding. (3) Epidemiology outcomes: compare malaria case incidence in each larviciding arm (SIS and Conventional) to standard of care. (4) Calculate the incremental cost-effectiveness of switching from conventional to SIS-based larviciding.

Each arm consisted of 15 clusters located within a hotspot shehia (administrative unit) with a malaria case rate higher than 5 per 1000 in 2018 (Malaria Case Notification data). Clusters consisted of a core of at least 250 houses, determined by counting the number of houses (provided by the Zanzibar Water Authority as a vector dataset) within 100 × 100 m grid squares. The 2012 census estimates the average household occupancy rate at 4.5 people per house [34] and, therefore, each cluster core was assumed to have a population of at least 1125. A buffer of at least 1000 m was placed between cluster core areas to avoid contamination. Interventions took place in core and buffer areas, but outcomes were only measured in core areas. Clusters were allocated to a study arm (15 clusters per arm) using restricted randomization balancing for dominant land use type (irrigated rice paddy or mixed agriculture) between arms. No larviciding took place in the Control arm. Standard of Care took place in all arms including case management, IPTp and ITNs. Study cluster design was done with the open-source geographical information system (GIS) software QGIS 3.16 [35].

The spatial intelligence system (SIS) intervention arm consisted of mapping and identifying breeding sites using drone and smartphone technology. In the mapping phase, carried out in May 2021, drones surveyed the SIS clusters (total area of 87.2 km^2^ with a mean area of 5.8 km^2^ per cluster). The drone images were processed to form a continuous image using AgiSoft Metashape [36], and operators digitally extracted potential habitats from the imagery using Technology Assisted Digitising (TAD) [37]. The locations potential habitats were then uploaded to the commercial Zzapp Malaria platform (www.zzappmalaria.com), which has an online dashboard showing details of the intervention programme and its progress (location and description of mapped breeding sites, details of larvicide treatment history including time of treatment, the field operative and larval sampling records). During the larviciding implementation phase, NMCP managers used the dashboard to allocate resources (staff and larvicide commodities) and track the progress of the intervention programme. The Zzapp dashboard links to the Zzapp smartphone app, which is used by larviciding operatives to locate habitats on the ground, record their characteristics and whether they were treated with larvicide. All data is uploaded to the Zzapp dashboard in real-time.

In the conventional larviciding intervention arm the mapping phase consisted of ground-based mapping of conventional clusters (total area of 84.3 km^2^ with a mean area of 5.6 km^2^ per cluster). This provided a paper-based inventory of potential malaria vector breeding habitats, including the coordinates of mapped sites provided by a hand-held GPS device. Conventional mapping was done by local community members, supported by members of ZAMEP’s entomology team. NMCP managers used the maps to allocate resources, and by operatives to guide themselves to habitats for treatment. Habitat characteristics (e.g. size and type) and status of treatment larvicide were recorded using a paper-based system. See the Additional file 1: Appendix (p 1) for details. In the conventional arm, mapping was initiated at the start of May 2021 with a second mapping survey at the end of May 2021 to refine the mapping data (adding missed sites and visiting areas that were not covered in the original survey).

In both the SIS and conventional arm, water bodies were treated every three weeks by local community workers over six rounds (18 weeks total) with the insect growth regulator Methoprene (low-toxicity Altosid: Central Life Sciences) using a motorized backpack sprayer (TGS30 TOMAHAWK 4). See the Additional file 1: Appendix (p 9) for details.

Although the ideal timing for LSM would have been during, or at the start of, the rainy season [38], the intervention began in June 2021 and was completed in October 2021, a typically dry period in Zanzibar. The timing of the intervention was dictated by Covid-related delays and logistical and financial issues imposed by finite project duration.

### Assessing mapping accuracy

To compare mapping accuracy between the two methods, simultaneous SIS and conventional mapping took place within eight 600 × 600 m areas randomly located across the conventional arm clusters (detailed in the Additional file 1: Appendix, p 14). The gold standard for mapping accuracy was provided by a systematic ground truth survey carried out by experienced field entomologists whereby each of the eight areas were further split into a grid of 10 × 10 m squares and each grid square was visited and characteristics recorded, including its inundation status (surface pooling of water) and presence of *Anopheles* larvae using a dipping strategy (ten samples using a standard 350 ml dipper). Where a grid square was inundated with water it was considered a ‘Treatment Unit’ (TU), i.e., a potential *Anopheles* breeding site that should be treated with larvicide. The ground truth survey was carried out in May 2021, coinciding with the timing of the SIS and conventional mapping surveys. Mapping accuracy for the SIS and conventional arms were quantified as the percentage of correctly identified (i) TUs and (ii) *Anopheles*-positive TUs, relative to those found in the ground-truth survey.

### Costing methods

Activity-based costing following the ingredients approach was used to estimate the financial and, separately, economic cost of SIS and conventional approaches to mapping and larvicide-treatment (Additional file 1: Appendix, p 19). Activities were identified and designated as start-up/regular and data on unit costs and quantity of different resource inputs consumed (e.g. personnel, equipment, consumables) were collected in custom designed spreadsheets by the Zanzibari team. Start-up activities were those that were deemed to only take place once in the life of a programme, whereas regular ones would be repeated each year. The costs of research activities (including the mapping accuracy assessment) were removed, and remaining cost data was analysed according to standard methods i.e., capital items were annualized by dividing by their useful life and by dividing by the useful life and (economic costing) applying a 3% discount rate. Start-up activity costs were further annualized over a five-year period with 3% discounting. Where activities (field worker training, management and supervision visits) were conducted jointly for both arms, 50% of their costs were allocated to each arm. All costs are reported in 2021 US$.

Cost data were summed to calculate the total costs of mapping related and, separately, larvicide-treatment related activities by arm. Unit cost per metre squared mapped were calculated by dividing total mapping related activity costs by the number of square metres separately, by arm. Cost per person protected was calculated by summing mapping and larviciding related activities and dividing by number of people living in the areas covered. Since the number of people living in the SIS arm was higher than in the conventional arm this was done firstly using unadjusted population estimates and then repeated using the mean population in the SIS and control arms.

Population data were estimated for 2021 by calculating household occupancy rate (4.5 people per house) using data from the Republic of Tanzania 2012 census (including projected population growth rates of 2.8% per year for Unguja) [34] and multiplying this by the number of households located in the study arms based on a spatial inventory (point vector) of buildings across Unguja obtained from the Zanzibar Water Authority.

### Malaria case incidence and cost-effectiveness analysis

Malaria cases recorded through the MCN surveillance programme during the study period were included in the analysis if their GPS data showed they resided in a study cluster. Case numbers and cluster population estimates (calculated using the approach described above) were used to estimate malaria case incidence in the study clusters. Cases were classified as local or imported by Malaria Surveillance officers, based on a series of travel questions asked to the patient. Cases classified as imported were not included in the analysis. Cases were allocated to a study arm where their coordinates (location of household) intersected a study cluster using QGIS 3.16 [35]. Only cases in the core areas of the clusters were included in the analyses.

The incremental cost per malaria case, malaria death and disability-adjusted life years (DALY) averted was estimated for SIS larviciding and compared to estimates for conventional larviciding using cost data and malaria case incidence from the MCN system (described above). Malaria cases averted in the SIS versus conventional arm were calculated and used to calculate DALYs averted using standard methods, with no discounting or age-weighting using Tanzanian life expectancy tables and malaria DALY weights (see the Additional file 1: Appendix, p 21 for details of input variables). Parameter uncertainty was propagated using Monte Carlo simulation, drawing 10,000 samples from probability distributions chosen in accordance with established practice. Simulation was conducted in @Risk (v8.4.0, Palisade Company LLC).

### Sample size estimations

The number of clusters included in the study was based on sample size calculations to detect a reduction in malaria case incidence of 50% (with a control incidence of 5 cases per 1000 person years), assuming an average of 1000 people per cluster and a follow up time of 1 year. With 80% power and an alpha of 5%, 15 clusters per arm were required.

No sample size calculation was done for the cost analysis that aimed to capture all costs or for the cost-effectiveness analysis, which used the sample size estimate for malaria incidence (described above).

### Statistical methods

A paired t-test was used to compare the proportion of correctly identified TUs across the two mapping approaches. Additionally, mapping errors were grouped into those within 50 m of a village centre and those further than 50 m from a village centre. This was done to determine whether villages, and their associated characteristics (i.e. a greater number of buildings, greater tree canopy cover, increase in the number of small artificial water sources), had an effect on mapping accuracy.

Case incidence was calculated per 1000 person years at a cluster level using population estimates for the core area of each cluster. Case incidence was compared between each intervention arm and the control arm using t-tests on the cluster level incidence rates. Linear regression models were used to examine rate difference between arms adjusting for baseline incidence.

## Results

### Mapping accuracy

Of a possible 28,800 grid squares, 9324 were visited (32.4%) by the ground-truth survey team for the mapping accuracy study. Missing grid squares were due to accessibility issues either due to extensive flooding, dense scrub/undergrowth or due to squares being within private property. A total of 181 TUs were recorded, with 21 testing positive for the presence of *anopheles* larvae.

The SIS approach correctly identified 125/181 (69%) of TUs identified in the ground truth survey compared to 86/181 (48%) correctly identified by the conventional approach (Table 1). In terms of *Anopheles*-positive TUs, the SIS approach correctly identified 18/21 (86%) compared to 11/21 (52%) for the conventional approach. Across the eight sites, the SIS approach was significantly better than conventional at identifying TUs (mean % correct per site: SIS = 60% (95% CI 32 to 88%, p = 0.02), conventional = 18% (95% CI − 3 to 39%)). There was no significant difference in the two approaches in terms of identifying *Anopheles*-positive TUs (mean per mapping accuracy site: SIS = 80% (95% CI 48 to 112%), p = 0.31, conventional = 43% (95% CI 4 to 83%).

**Table 1:** Comparison of Treatment Units (TUs: 10 × 10 m grid squares inundated with water) identified by the SIS and Conventional mapping approaches, compared against TUs found in the ground-truth survey

	All potential breeding sites					*Anopheles* positive breeding sites				
Site	Ground truth TU	SIS TU	% correct	Conv TU	% correct	Ground truth TU	SIS TU	% correct	Conv TU	% correct
Site 1	5	0	0	0	0	0	0	–	0	–
Site 2	36	9	25	6	17	2	0	0	2	100
Site 3	13	8	62	0	0	1	1	100	0	0
Site 4	83	71	86	69	83	6	6	100	6	100
Site 5	11	11	100	0	0	5	5	100	0	0
Site 6	6	1	17	0	0	0	0	–	0	–
Site 7	2	2	100	0	0	2	2	100	0	0
Site 8	25	23	92	11	44	5	4	80	3	60
**Mean**	**22.6**	**15.6**	**60**	**10.8**	**18**	**2.6**	**2.3**	**80**	**1.4**	**43**
**Total**	**181**	**125**	**69**	**86**	**48**	**21**	**18**	**86**	**11**	**52**

Bold values simply refer to the mean and total values for each column, making the cells distinct from the rest of the data

**Fig. 2:**
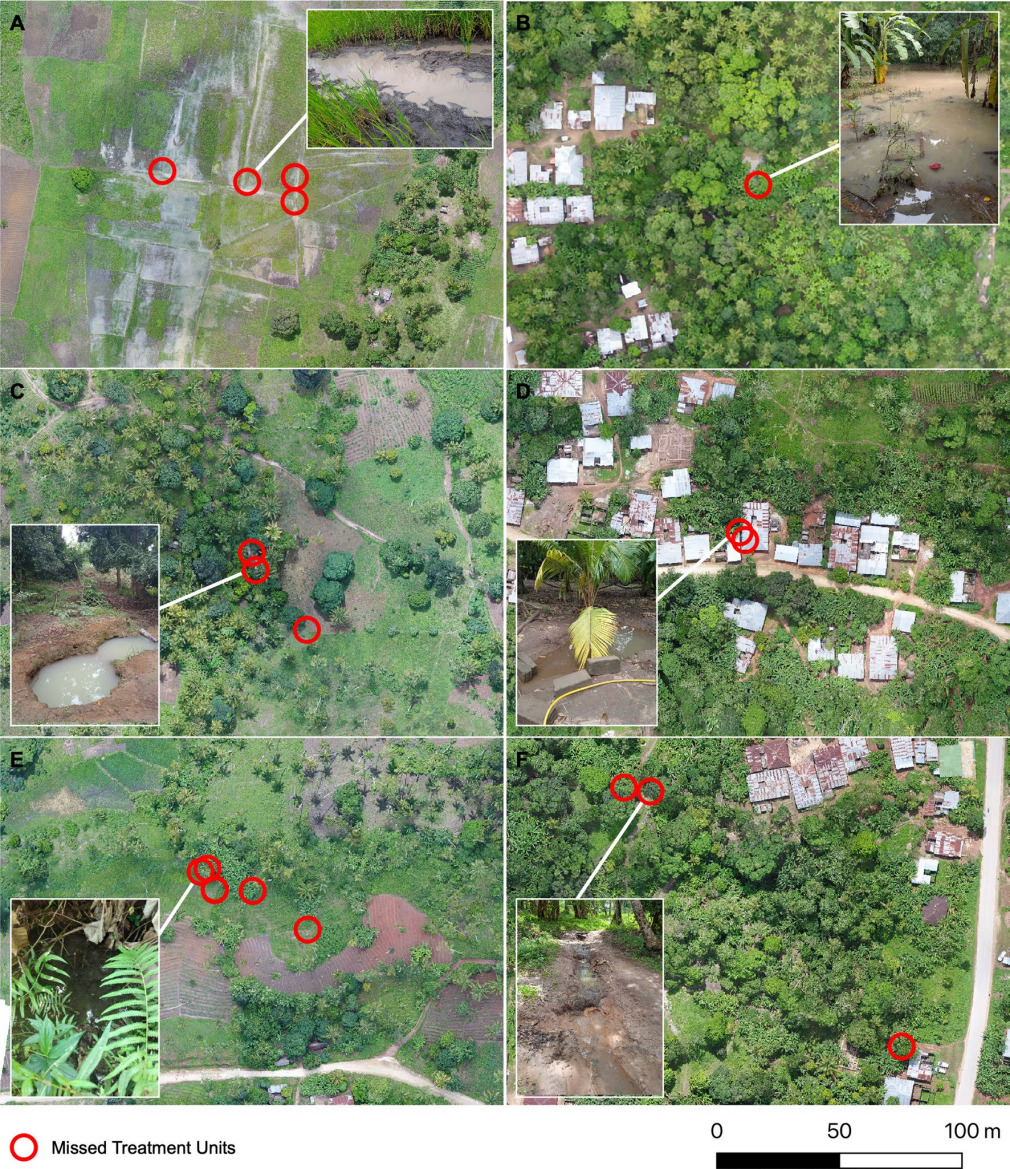
Example Treatment Units (TUs) that were missed by conventional mapping: **A** Area of extensive flooding, **B** dense thicket and forest **C** isolated pools and SIS mapping: **D** obscured by buildings, **E** small water bodies < 2 m and **F** water bodies underneath tree canopies

For both mapping approaches, the greatest proportion of missed TUs were located within 50 m of the centre of a village (Table 2). TUs were found across a range of habitat types: standing water in tracks, swamps, stream channels, rice paddies, isolated pools of water and construction pits (Table 3). The SIS approach out-performed conventional mapping across all habitat types, bar agriculture, with particularly high results for TUs located in rice paddies (97%, n = 71 of TUs correctly mapped) and swamps (100%, n = 10). By comparison, conventional mapping performed relatively poorly, particularly for more discrete water sources such as isolated ponds in relatively remote locations (27%, n = 6) and pools of water in vehicle/footfall tracks (28%, n = 8). For conventional mapping, the main reason why TUs were missed was because of difficulties in accessing the location due to extensive flooding or dense forest and thicket (49%, 47 out of 95 missed TUs) (Fig. 2). The majority (75%, 43/57) of TUs missed by the SIS approach were less than 2 m wide. Additionally, 40% of TUs (n = 23) missed by the SIS approach were located under tree canopies.

**Table 2:** Proportion of Treatment Units (TUs: 10 × 10 m grid squares inundated with water) missed by the SIS and Conventional mapping approaches, summarized by distance from a village centre (more than or less than 50 m from a village centre)

Distance	GTS TU count	SIS			Conventional		
		TUs Found	TUs Missed	% Missed	TUs Found	TUs Missed	% Missed
< 50 m from village	53	23	30	56.6	14	39	73.6
> 50 m from village	128	102	27	21.1	72	57	44.5
All	181	125	57	31.5	86	96	53.0

**Table 3:** Number and percentage of Treatment Units (TUs: 10 × 10 m grid squares inundated with water) and *Anopheles* positive TUs missed by the SIS and conventional mapping approaches, summarized by habitat type

Type	TUs			Anopheline positive TUs		
	GTS	SISs %	Conv %	GTS	SIS %	Conv %
Agriculture	22	4 (18)	6 (27)	2	0 (0)	2 (100)
Construction	2	0 (0)	0 (0)	–	–	–
Fringe	6	4 (67)	3 (50)	–	–	–
Home	17	3 (18)	0 (0)	–	–	–
Isolate pond	22	14 (64)	6 (27)	–	–	–
Rice paddy	73	71 (97)	57 (78)	11	10 (91)	5 (45)
Swamp	10	10 (100)	6 (60)	2	2 (100)	2 (100)
Vehicle/walking track	29	19 (66)	8 (28)	1	1 (100)	0 (0)

### Operational LSM

**Fig. 3:**
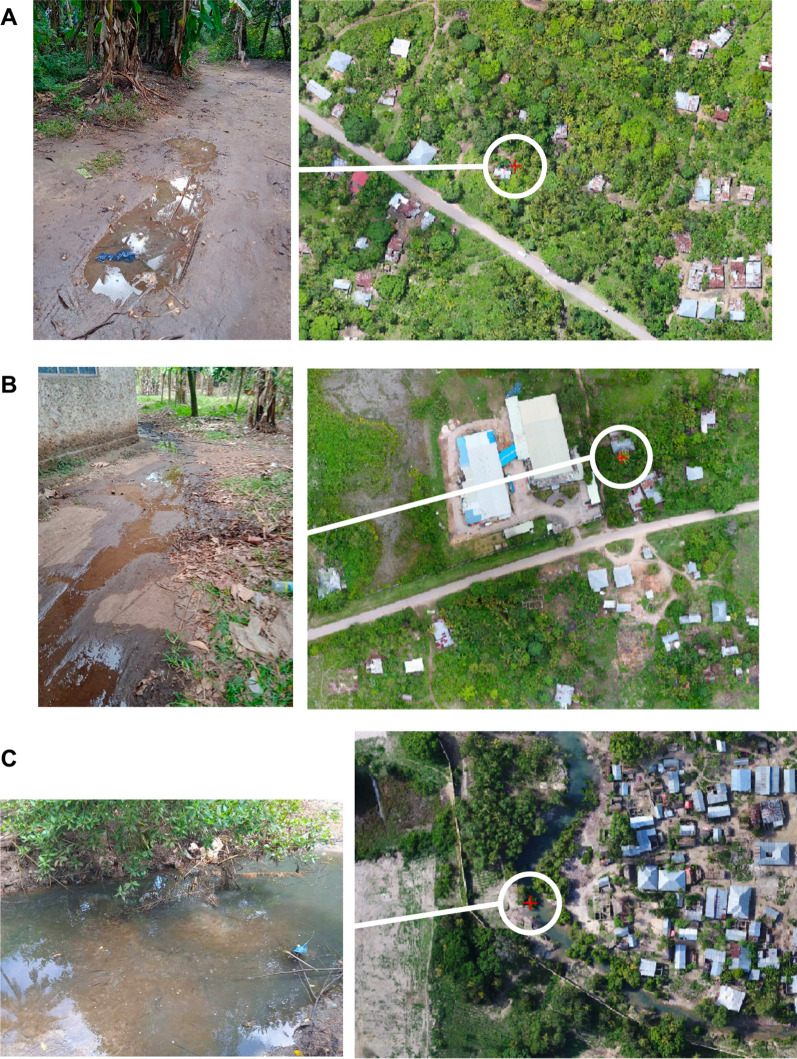
Examples of *Anopheles* larval habitats that were missed by the drone mapping process but recorded by field operatives within the SIS arm. **A**: Small (< 3 m) breeding site on a track underneath dense forest canopy; **B**: Small (< 1 m wide) breeding site obscured by a structure; **C**: Medium (~ 5 m wide) breeding site within a stream channel obscured by riparian tree canopy

A total of 382 potential breeding sites were mapped in the conventional arm. Mapping in the SIS arm identified a total of 1122 potential breeding sites of which 880 were directly mapped using the drone imagery and the remaining 242 (22%) were mapped by SIS larviciding operators in the field whilst navigating on the ground to drone-mapped sites using the Zzapp Malaria system. Examples of water sources missed by the drone that testing positive for the presence of anopheline included small, isolated water bodies obscured by building structures, tree canopies and habitats in sections of stream channels obscured by overhanging tree canopies (Fig. 3).

### Costing results

Before annualization (i.e. before adjusted for capital expenditure items to account for their value over a useful life with duration greater than 1 year), the total economic cost of SIS was $96,164 compared to $70,527 for conventional LSM with cost of start-up activities accounting for 36.0% and 2.2% for SIS and conventional LSM, respectively. After annualizing, start-up activity costs over 5-years (assumed useful life), SIS economic cost was $63,700 (start-up 11.9%) compared with $65,131 (start-up 0.5%) for conventional (Additional file 2: Table S1).

Mapping related activities (mapping and mapping management and supervision in both start up and regular phases, and mapping specific training at start-up, and routine and specialist app training in the SIS arm) accounted for $38,746 (60.8%) and $31,291 (48.0%) of SIS and conventional (annualized) costs, respectively (Additional file 2: Table S1). Annualized economic cost per km^2^ mapped were higher in the SIS than the conventional arm ($444.3 versus $271.3).

Intervention delivery activities (including start-up phase activities related to equipment procurement, and regular activities for six monthly larviciding rounds with supervision) accounted for $24,954 (39.2%) and $33,840 (52.0%) of annualized SIS and Conventional larviciding costs, respectively (Additional file 2: Table S1). Intervention delivery costs were lower for all rounds in the SIS arm than the conventional arm with this difference being driven by lower personnel time costs i.e. over six treatment rounds the SIS arm required 1102 h worked compared to 1603 for the conventional arm (see Additional file 1: Appendix, p 28).

Considering the costs of all activities in both start up and regular phases, and the adjusted mean population for the total intervention area, the (annualized) unit cost per person protected was similar for the SIS versus the conventional arm ($1.96 versus $2.00). Simulated mean costs per km^2^ of land covered with larviciding, and, per person protected were lower for SIS than Conventional but the 90% credible intervals overlapped (Table 4). Point estimates of the incremental cost per km^2^ and person protected per year with 6 rounds of larviciding of switching between conventional and SIS larviciding were both negative (i.e. suggesting costs savings) although the 90% credible interval for cost per km^2^ came very close to zero and for per person crossed zero.

**Table 4:** Simulated cost per km^2^ covered, person protected per year by arm and cost-effectiveness of SIS versus conventional larviciding per case, death and DALY averted (US$)

Arm	Cost per unit	Mean cost (and 90% credible interval) US$
SIS	km^2^ of land covered with intervention	739.44 (721.638 to 761.823)
	Person protected per year (6 rounds of larviciding)	1.82 (1.776 to 1.875)^§^ 3.69 (0.319 to 15.116)^§§^
Conventional	km^2^ of land covered with intervention	795.77 (744.39 to 859.05)
	Person protected per year (6 rounds of larviciding)	2.26 (2.120 to 2.446)^§^ 3.94 (0.342 to 16.273)^§§^
SIS v Conv	Incremental cost per km^2^ of land covered with intervention	− 56.33 (− 121.97 to -0.976)
	Person protected* per year with 6 rounds of larviciding	− 0.278 (− 13.39 to 12.15)
	Case averted	1430.77 (-5,869.81 to 5,839.23)
	Death averted	357,438.15 (− 2,693,340.55 to 2,698,272.32)
	DALY averted (SIS v conventional)	6,209,51 (− 47,814.20 to 47,634.36)

^§^Uses a fixed estimate based on actual number of people protected

^§§^Uses a range of people protected based on population density per km^2^ in rural areas from the 2021 Zanzibar census data

Uses ^§§^ as above, i.e. the cost per km^2^ covered with each type of SIS divided by population density per km^2^ from Zanzibar for rural areas only. Including more densely populated urban areas would improve this substantially but we did not test drones in this type of area

### Intervention impact and cost-effectiveness

During the baseline year (1^st^ June 2020 to 31^st^ May 2021) there were 384 locally acquired malaria cases recorded in the Malaria Case Notification (MCN) system in the study area, giving an overall incidence of 4.19 (95% CI 3.06–5.32) cases per 1,000 person-years. This varied slightly by arm, with the highest incidence measured in the conventional arm as 5.61 (95% CI 2.95–8.26) (Additional file 2: Table S2).

In the follow up period (1st June 2021–31st May 2022), there were 102 locally acquired malaria cases reported in the study area (overall incidence of 1.10 (95% CI 0.80–1.39) cases per 1,000 person years). Incidence had reduced in all three arms. There was no difference between either intervention arm and the control arm in terms of malaria case incidence (p > 0.05 for both comparisons, Additional file 2: Table S2). Adjusting for baseline incidence did not change the result.

The adjusted rate difference between the conventional larviciding arm compared to the SIS arm was -0.15 (95% CI − 0.91–0.61), p = 0.693.

In the base case analysis, the incremental cost-per case, death and DALY averted of switching from conventional to SIS larviciding were all positive, however the 90% credible intervals were wide and range from negative to positive indicating a high degree of uncertainty as to the ratio of cost and effects of switching from conventional to SIS approach (Table 4).

**Fig. 4:**
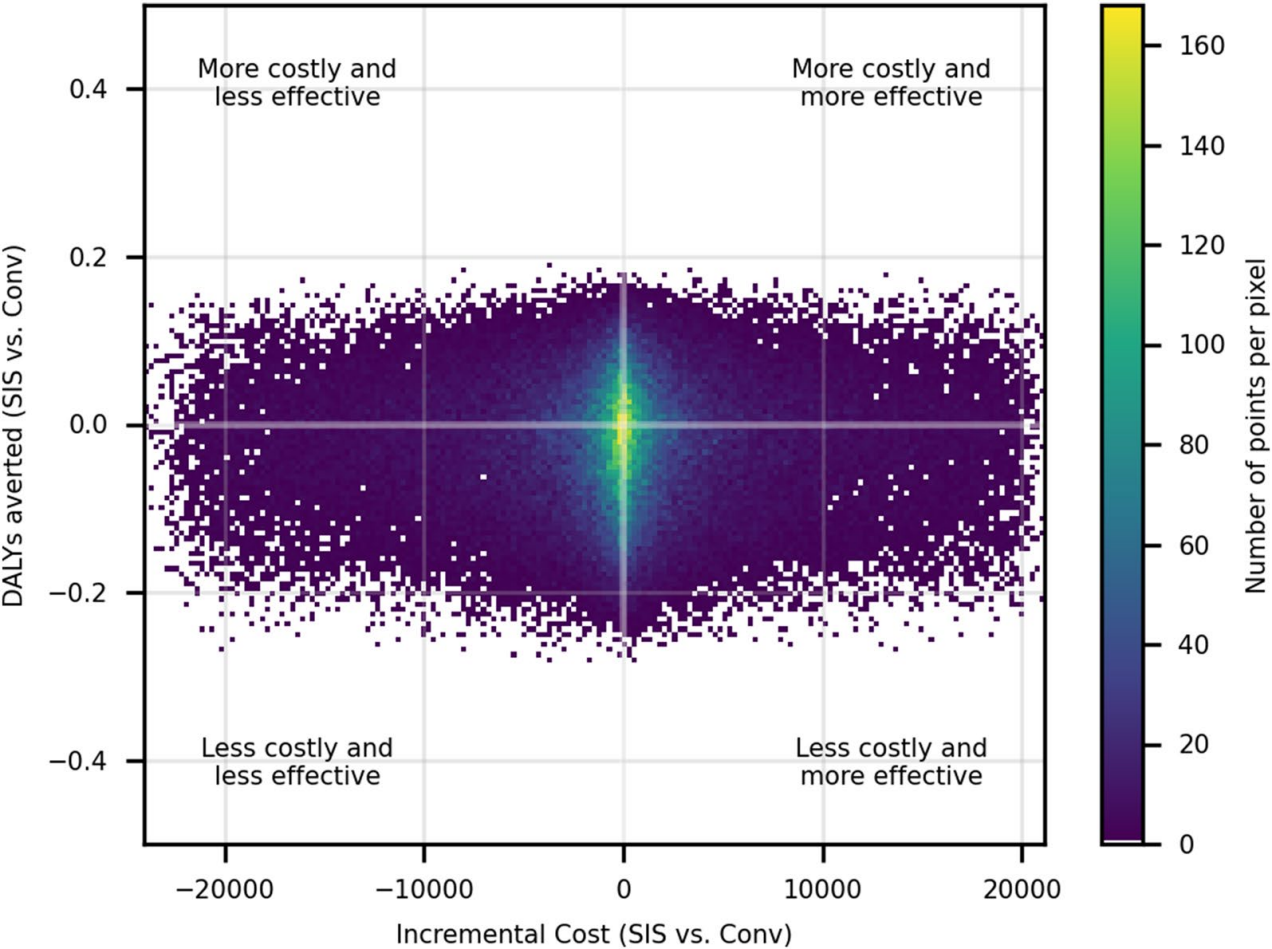
Cost-effectiveness plane comparing incremental cost per DALY averted of SIS larviciding versus conventional larviciding

The results of the Monte Carlo simulation were plotted on a cost-effectiveness plane and show that a large number of simulated outputs fall in each quadrant with the densest part of the plot clustered around the origin (Fig. 4), suggesting that SIS is likely to provide similar health benefits at similar costs compared to the conventional arm.

## Discussion

### Epidemiology

Malaria case incidence was substantially reduced in the follow up year, with a more than 60% reduction in incidence in all arms compared to the baseline year. Incidence was much lower than was assumed in the original power calculations, which could have contributed to the null result measured in the trial. The reduced incidence could have been caused by targeted interventions, such as IRS, which simultaneously occurred across many of the study clusters but may also have been an impact of the Covid pandemic, which is likely to have reduced the numbers of infected people reporting to health facilities and resources for subsequent follow up. The lack of epidemiological impact of the larviciding may also be due to the timing of the intervention, that took place over the dry season and, therefore, may have diluted its impact. Timing the intervention to target the peak rainy season may have seen more impact on malaria cases, however due to Covid-related delays and logistic issues, that was not possible in this study [38].

### Economics

The cost per area covered and per person protected were similar for SIS and Conventional larviciding in both the cost analysis and cost simulations. Simulated cost per person protected per year was $3.69 ($0.32 to $15.12) for conventional and $3.94 ($0.342 to $16.27) for SIS larviciding. These costs are comparable with larviciding in other contexts which provided broadly similar cost estimates ranging from $1.33–2.53 in Kenya (14), $1.64 in Tanzania [39], $0.95–1.29 in Burkina Faso [14] and $27.70 in Malawi [22] (all in 2021 USD).

Start-up costs were higher for SIS than conventional LSM, but routine implementation costs were lower for SIS than conventional, with more than half of the SIS-based start-up costs being attributed to a community sensitization and engagement programme that was commissioned to ensure trust and acceptance of drones [40]. Community engagement was carried out by a locally-based independent consultancy but in future, it might be possible for NMCPs to conduct sensitization potentially reducing start-up costs.

Notably, personnel costs were lower in the SIS versus the conventional arm highlighting potential efficiency gains offered by drone technology. Given that labour is often the highest recurrent cost in operational programming, reductions in this resource could aide sustainability whether workers are paid or voluntary.

Monte Carlo simulation was conducted to compare the incremental benefits (in DALYs averted) of switching from a conventional to SIS based approach, using available cost and outcome (change in malaria incidence). Given that both costs and effects of SIS and conventional LSM were similar, the simulated cost-effectiveness was characterized by a large degree of uncertainty with many simulations falling in each quadrant of the cost-effectiveness plane and the densest area of the plot was around the origin. This suggests that SIS is likely to provide similar outcomes at similar costs when compared to conventional larviciding. Estimates of cost-per DALY averted cannot be compared with other estimates of the cost-effectiveness of larviciding in the literature since this study compares SIS with conventional larviciding, rather than others that commonly compare larviciding to no larviciding. Given the known issues with timing of the intervention, and the lack of evidence of epidemiological impact of any larviciding compared to none, this analysis was not undertaken. It is likely that this analysis would have shown that larviciding was more costly and similarly effective than standard of care (top left-hand quadrant of the cost-effectiveness plane in Fig. 4).

As with any malaria prevention intervention, a substantial amount of uncertainty in the cost-effectiveness estimate arises from assumptions used in the DALY calculation particularly in relation to life years saved per death averted. Assumptions about the spatial scale of the intervention in relation to the humans protected within that space also leads to uncertainty. For larviciding this is particularly challenging as the magnitude of the heterogeneity in population density per km^2^ is likely larger than it is per house (for IRS) or per sleeping space (for ITNS). This presents a methodological challenge for the economic evaluation of vector control interventions, such as larviciding, spatial repellents, and gene drive strategies, which are designed to protect populations beyond the confines of a sleeping space or house structure. Tackling this will require expertise from economists adept at working in vector control, and close scrutiny to ensure that assumptions are realistic and subject to robust sensitivity/uncertainty analysis to ensure that results are comparable with other malaria vector control interventions.

Following ZAMEP standard operating procedures this study employed the services of community volunteers to disseminate larvicide in potential mosquito larval habitats. However, this approach becomes challenging in areas with extensive habitats like rice paddies [25]. Commercially available systems for spraying larvicide from a drone platform also exist, providing a labour-saving solution for treating such habitats. In this study, water sources associated with rice paddies made up 19% of all potential mosquito larval habitats mapped and they accounted for 2% of *Anopheles* positive sites. Evaluation of the use of drone-spraying technology for treating other habitat types (particularly those with safety and logistical factors such as habitats close to trees or people’s homes), together with their associated costs, needs to be made before clear recommendations can be made to NMCPs about the use of drones to disseminate larvicide.

### Habitat mapping approaches

If new tools are to be integrated into vector control strategies, they need to be owned and operated by those delivering interventions. This is particularly pertinent with new technological equipment (including drones) that have historically been deployed from the global North into communities in the global South [41]. This study demonstrated that drone and smartphone technology can be owned and operated by the NCMP in Zanzibar through the collaboratively designed SIS framework, providing a model for other vector control programs.

The key benefit of using the SIS approach was that drone imaging technology offered coverage of a wide area meaning that a greater number of potential mosquito larval habitats can be identified and mapped compared to a conventional ground-based mapping approaches that faces a number of logistical challenges. For example, conventional mapping tended to miss habitats located in areas that were difficult to access due to extensive flooding, dense forest/thicket or where there was a lack of path and tracks. The ground-truth survey, used for assessing the accuracy of the two mapping approaches, also faced these logistical challenges meaning that the TUs identified were relatively accessible and probably underrepresents habitats in less accessible locations, particularly in areas with extensive inundation that are difficult to access on foot, but are readily mappable with drone imagery. As such, it is possible that the evaluation undertaken in this study is underestimating the value of the drone imagery for mapping habitats compared to ground-based approaches. In this respect, the drone technology offers LSM implementers a distinct advantage: enabling habitat surveys to be carried out over wider areas largely independent of landscape conditions.

The majority of potential mosquito larval habitats identified within SIS clusters were mapped using the drone imagery and the TAD mapping process [37], yet nearly a quarter of sites (22%) were added to the spatial inventory by field larviciding operatives. In village locations, water sources were often missed by the drone because they were obscured by tree canopies or structures (Fig. 3A and B). Given their proximity to people’s homes, these water sources represent potentially important malaria vector habitats and future operational LSM programmes supported by drones should consider how these water sources might be mapped. Conventional mapping also performed relatively poorly in villages due to difficulties in accessing private land. As such, it is recommended that the SIS drone-based surveying should be combined with a community-led habitat mapping scheme. To this effect, a community engagement approach is proposed that develops people’s understanding of vector habitats, building towards a household level survey of localized water sources and subsequent treatment plan where drone and conventional mapping is less effective. This approach would have broader benefits where explicit community participation leads to a higher potential for vector control uptake, sustainability and success [42, 43]. In terms of drone usage, whilst a pre-deployment study identified strong community support for the use of this technology in Zanzibar [40], community-based drone mapping is not necessarily recommended, as surveys of this nature require permits and approval that is more appropriately obtained by the NMCP.

Often, potential mosquito larval habitats missed by the SIS approach were in close proximity to mapped habitats. For example, a series of pools of water belonging to a stream were mapped by the drone but sections of the stream were missed because they were underneath the canopy of trees (Fig. 3C). Similarly, in rice paddy environments, inundated fields could readily be identified in the drone imagery, but these were characterized by 100 s of small, discrete water body features that were laborious to digitize leading to missed sites (Table 3). However, these types of errors could be addressed by adapting the SIS approach in two ways: (1) single sets of coordinates can be digitized marking the location or access point for relatively large, inundated areas such as rice paddies and lakes. (2) Digitizing operators can be trained to identify basic hydrological features, such as lines of trees that are indicative of streams and rivers and digitize these features using a simple polyline. These two recommendations will speed up the mapping process whilst ensuring sufficient information for field operatives to locate and treat the habitat.

### Study limitations

Whilst the impact of the larviciding intervention was not the main aim of this study, it would have been preferable to collect entomological data to assess the impact of larviciding on the vector population. Evaluating the impact of a growth regulator, such as Methoprene used in this study, requires a longitudinal survey of adult vector densities. However, this was prohibitively expensive, particularly as vector densities are low in Zanzibar (based on data provided by ZAMEP), therefore requiring a large number of sample sites to power a difference between intervention and non-intervention study arms.

As described, ground-based mapping of habitats faces a number of logistical challenges, particularly where there is extensive flooding, thick undergrowth or forest, or limited access to private property. Future work may consider shadowing ground-based mapping teams, recording (using a GPS) the area that was covered, enabling a comparison of coverage (i.e. the proportion of area where mapping effort took place) between conventional and SIS mapping approaches.

This study was focussed on evaluating habitat mapping approaches in the context of operational larviciding. As such, field spray operatives were only required to make relatively quick in-situ observations of larval stage and genus, rather than extract larval samples for determining species. Nevertheless, collecting this data may have revealed habitat type preferences as well the overall vector species composition, helping to inform future LSM campaigns in Zanzibar.

## Conclusions

For the first time, this study evaluates the practical use of drones and smartphones (as part of the integrated SIS approach) in supporting operational larviciding for malaria vector control. This study has demonstrated that drones are more accurate than conventional ground-based surveys in mapping habitats. Moreover, deploying this technology does not appear to increase, and may reduce, the costs associated with both mapping and treating potential malaria vector habitats. As such, the combined use of drone and smartphone technology may represent an important tool for NMCPs that plan to implement larviciding. The developed drone mapping approach was not designed to be malaria vector specific and, therefore, there is potential for the developed system to be used in controlling vector mosquito habitats for other diseases, such as dengue fever, West Nile virus and Rift Valley fever.

### Supplementary Information

**Additional file 1: ** S1 Details of the habitat mapping protocol used in the conventional arm. S2 The larviciding protocol used in both intervention arms. S3 Details of the mapping quality study data collection and analysis. S4 Details of the costing methods. Table 1 S5 Summary of the input variables used in the cost-effectiveness simulation modelling. Table 2 S6 Summary of the costs related to the intervention.

**Additional file 2: Table S1**. Unannualized cost and annualized economic and financial costs by study arm (SIS or Conventional) phase (start up or regular) and activity (US$). **Table S2**. Summary of case incidence data and rate difference between the baseline year and the follow up period.

## Data Availability

Economic cost data can be accessed in the Supplementary Materials.
