# Supplementary material for: Cost and quality of operational larviciding using drones and smartphone technology
Source: Malar J. 2023 Sep 27;22:286. doi: 10.1186/s12936-023-04713-0 (PMC10523724; doi:10.1186/s12936-023-04713-0)
Supplement: Supplementary file 1 — Additional file 1: S1 Details of the habitat mapping protocol used in the conventional arm. S2 The larviciding protocol used in both intervention arms. S3 Details of the mapping quality study data collection and analysis. S4 Details of the costing methods. Table 1 S5 Summary of the input variables used in the cost-effectiveness simulation modelling. Table 2 S6 Summary of the costs related to the intervention. [file 12936_2023_4713_MOESM1_ESM.docx]

**Supplementary Appendix**

**Conventional Mapping Protocol**

# Goal: To map potential mosquito breeding habitats in all conventional larviciding clusters in order to plan efficiently Larval Source Management operations to be carried out in all the districts from June 2021 to October 2021 (5-month period).

**Specific objectives:**

- Determine the spatial and temporal changes in available aquatic habitats in all the Conventional Larviciding (CL) clusters between June 2021 to October 2021; this time will allow you to understand the breeding habitats dynamics between rainy and dry seasons
- Determine the changes in colonisation of these aquatic habitats by *anopheles* and *culicine* mosquitoes
- **At the end of the first round of mapping select two sentinel sites** in each of the CL clusters to survey the density of larvae per dip and monitor the changes over time;
- Identify the variability in *anopheles* habitat types over space and time

This information is necessary to help target larviciding measures and help determine whether we can successfully control the aquatic stages of the vector with larviciding. We should be able to compare the proportion of available aquatic habitats that are colonised by mosquitoes, the proportion of habitats that contain late instar larvae, the mosquito density in sentinel sites, before and after the larviciding intervention and see whether there is a reduction in the number of colonised habitats and larval densities due to larviciding.

**Community sensitization**

It is mandatory to inform and gain consent from the administration, community leaders and the community members before any mapping and larviciding can take place in the intervention areas. Community members are usually very concerned about pesticides applied in their houses or environment. There is usually the fear that pesticides applied on water could affect human beings or livestock.

**Communities should be visited and informed about the planned activities**. Community leaders need to be informed and with their help community meetings need to be held. Any questions and concerns of the community need to be answered to the best of your knowledge. Questions that cannot be answered immediately need to be discussed with ZAMEP management and information brought back to the community. Everyone involved in the sensitisation process should have the same message to the population. Therefore, a leaflet and a frequently asked questions fact sheet (see appendix) will be distributed during the sensitization meetings to those chairing the meeting and anyone else in the community who can read and is interested.

**Materials**: 1 backpack, 1 GPS and spare batteries in plastic bag (or tablet), 1 Standard Dipper with handles, 1 small dipper, 2 plastic pipette, 1 map, 1 copies of habitats definition, data recording forms in plastic holder, 4 sheets of plain paper, 1 clip board, 2 pencils, enough drinking water, hat, rain coat, boots

**Methods:**

The mapping will take place in all the CL clusters, in areas inhabited by people up to a 5 km radius around human settlements, if further away there is an extensive land mass, forest, river or other features with no human settlements.

To identify mosquito larval habitats, it is essential to be systematic and check all possible breeding places (any stagnant water body), even those that are hard to reach. This enables determination of the types of sites most likely to harbour the aquatic stages of *anopheles* mosquitoes.

Potential breeding sites include:

- **River edges**
  ⇒ here the larvae are normally associated with vegetation or edges, where sampling should take place on the edges and in the main water body
- **Small pools, puddles, foot-prints, tyre tracks**
  ⇒ the entire surface of water should be examined for mosquito larvae.
- **Streams**
  ⇒ should be searched at edges, where there is vegetation and the water moves slowly and forms stagnant pools
- **Ponds**
  ⇒ larvae can occur in vegetation around the edges
- **Special sites like wells and cemented pits and basins to collect water**
  ⇒ the entire surface should be examined for larvae in case of sites not exceeding 10m^2^, in large surface areas larvae will be mostly associated with edges and vegetation. Special nets may have to be used to collect larvae from deep wells.
- **Agriculture areas** (ricefields, irrigated lands, etc)
- **Swamps, mangroves**

Mosquitoes are adapted to a large variety of aquatic habitats and can be found in nearly all types of stagnant water bodies, a precise mapping is therefore essential. Even tin cans, small plastic containers and other garbage that collects water can provide suitable habitats for mosquito breeding, though these container type habitats are preferred by *aedes* mosquitoes not involved in malaria transmission, but they should be removed/treated.

Preferred (but not restricted) sites where *anopheles* (malaria vector) larvae can be found:

- sunlit water bodies or the sun-exposed area of a water body,
- edges of water bodies,
- around low vegetation e.g. grass tuffs,
- around swimming debris and leaves,
- in-between floating vegetation
- except in very small sites, *anopheles* larvae are usually NOT evenly distributed over the entire surface area.

# The dipping technique

- The most common and easiest technique to investigate the presence of mosquito larvae and their density is dipping
- A dipper can vary in shape and size, including small pans, soup ladles and photographic dishes can be utilized
- A dipper (see ‎Figure 1) should be light in colour inside to see the larvae easily, the amount of water you dip should be known if you want to measure densities per volume.


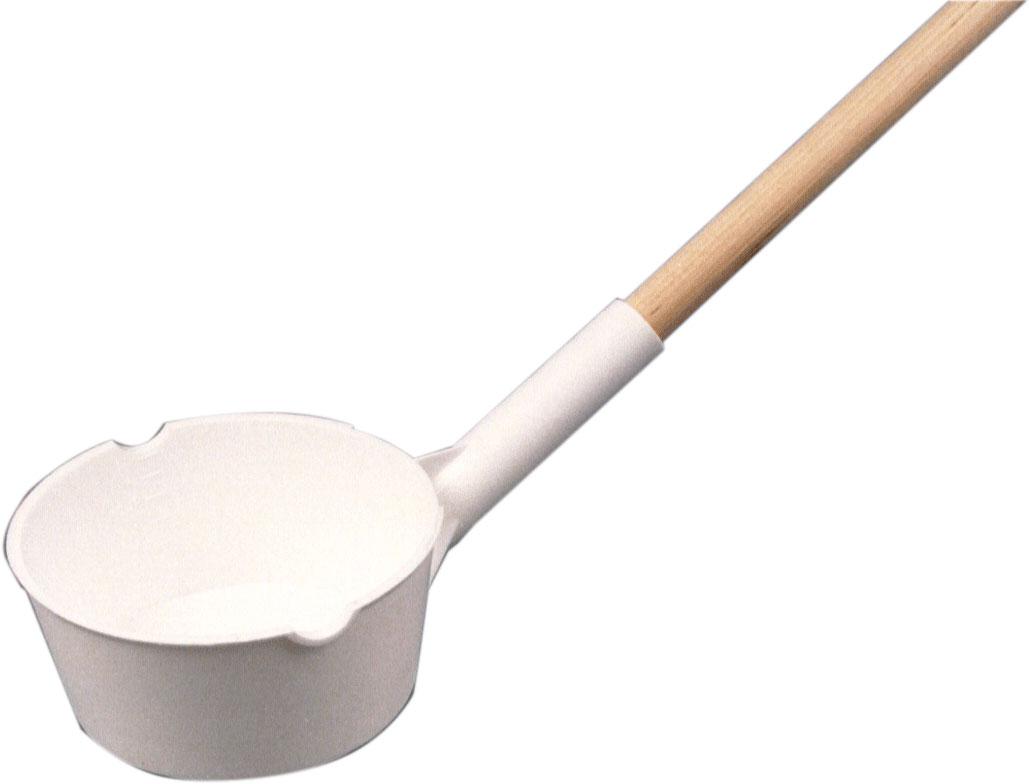


1. A Standard Mosquito dipper

# Methods of use:

- Lower the dipper gently in an angle of 45° just below the surface so that water flows in with any larvae that might be present;
- take care not to disturb the water too much otherwise larvae will swim downwards and may not be collected. If sampling in open water, wait 3 minutes before dipping;
- when lifting the water, take care not to spill the water containing the larvae and pupae;
- hold the dipper steadily until larvae and pupae rise to the water surface in the dipper (can take several minutes, especially with older instars).
- If you need to identify whether the stagnant water body is a mosquito breeding habitat at the moment of the survey identify and record the presence or absence of *anopheles* and culicine larvae separately and return them to the water (take care not to disturb the water surface too much).
- If you need to measure larval densities per dip count all larvae present, distinguish between genera (*anopheles* and culicine) and instars (early =L1-L2 and late =L3-L4, pupae), return larvae back. **DO NOT THROW** the water back into the breeding place otherwise you will disturb any larvae and pupae for further sampling.

**REMEMBER** that *anopheles* densities are often quite low compared with other genera, and you have to extend your time and efforts to detect them! Furthermore: dipping pupae is extremely difficult because they are very sensitive and fast, the slightest disturbance and they disappear (dive), additionally they are even more clustered at one spot than larvae, therefore the number of pupae per dip might be underestimated.

Where there is dense, floating vegetation:

- Disturb water, causing immature to sink below the surface
- Clear away vegetation with the dipper and wait a few minutes for larvae and pupae to return to surface
- In clumps of vegetation (e.g. grass) press dipper into vegetation so that water flows into the dipper.

***Sampling extreme small habitats***

Small breeding sites like hoof prints where a dipper does not fit are difficult to sample. One can either collect with a small sieve, a spoon or a pipette: therefore, it can be helpful to stir the water with a stick to make it muddy and watch for the larvae and pupae to rise, because they are now easily to be seen against the muddy background.

***Landcover type***

The aquatic habitats that represent potential breeding habitats for mosquitoes can be distributed over the whole district. Semi-permanent and permanent aquatic habitats are characterised by vegetation cover. In the mapping we want to identify the dominant vegetation type that characterises the environment at large at the specific site ID.

*1. Grassland:* Areas where grassland dominates

*2. Agriculture:* Areas where agricultural fields dominate the surrounding.

*3. Bush:* Landcover that is dominated by bushes with many open areas.

*4. Forest:* Landcover type that is dominated by large trees and few open areas.

*5. Barren floodplains:* Areas in the floodplain of a river where there is no vegetation.

*6. Sedge:* Areas characterised by sedges (grasses that are dark green, have a hard, stem and either grow as singular spikes or tufts).

*7. Rice:* Landcover type when rice has been planted in the rice fields.

*8. Tall Reeds:* Landcover type that is dominated by tall reeds. Tall reeds are often characteristic for open floodwater areas and streams.

*9. Mangrove:* Mangrove trees characterise the area of the aquatic habitat mapped.

##### Aquatic habitat types to be distinguished in larval surveys

The following habitat types shall be distinguished:

*1. Edge of floodwater*
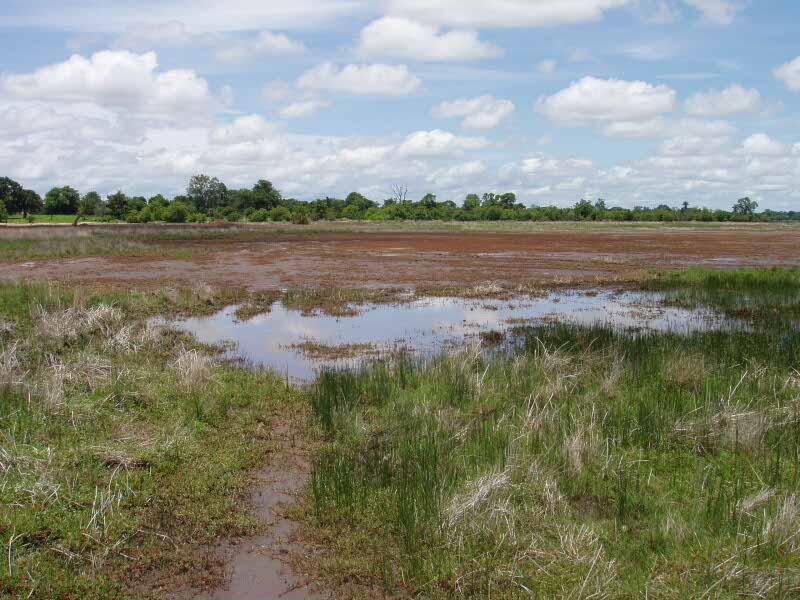


This is the landward edge of floodwater **in the floodplains**. The water will most of the time be shallow and might not always be a single big water body but might consist of a number of pools and puddles. The landward edge of the floodwater will often be associated with barren floodplains or floodplains characterised by grasses and sedges. It is always the first water body you find when entering the floodplains from the upland. The floodwater is in contrast to pools and ponds not a very discrete water body.

*2. Floodwater*


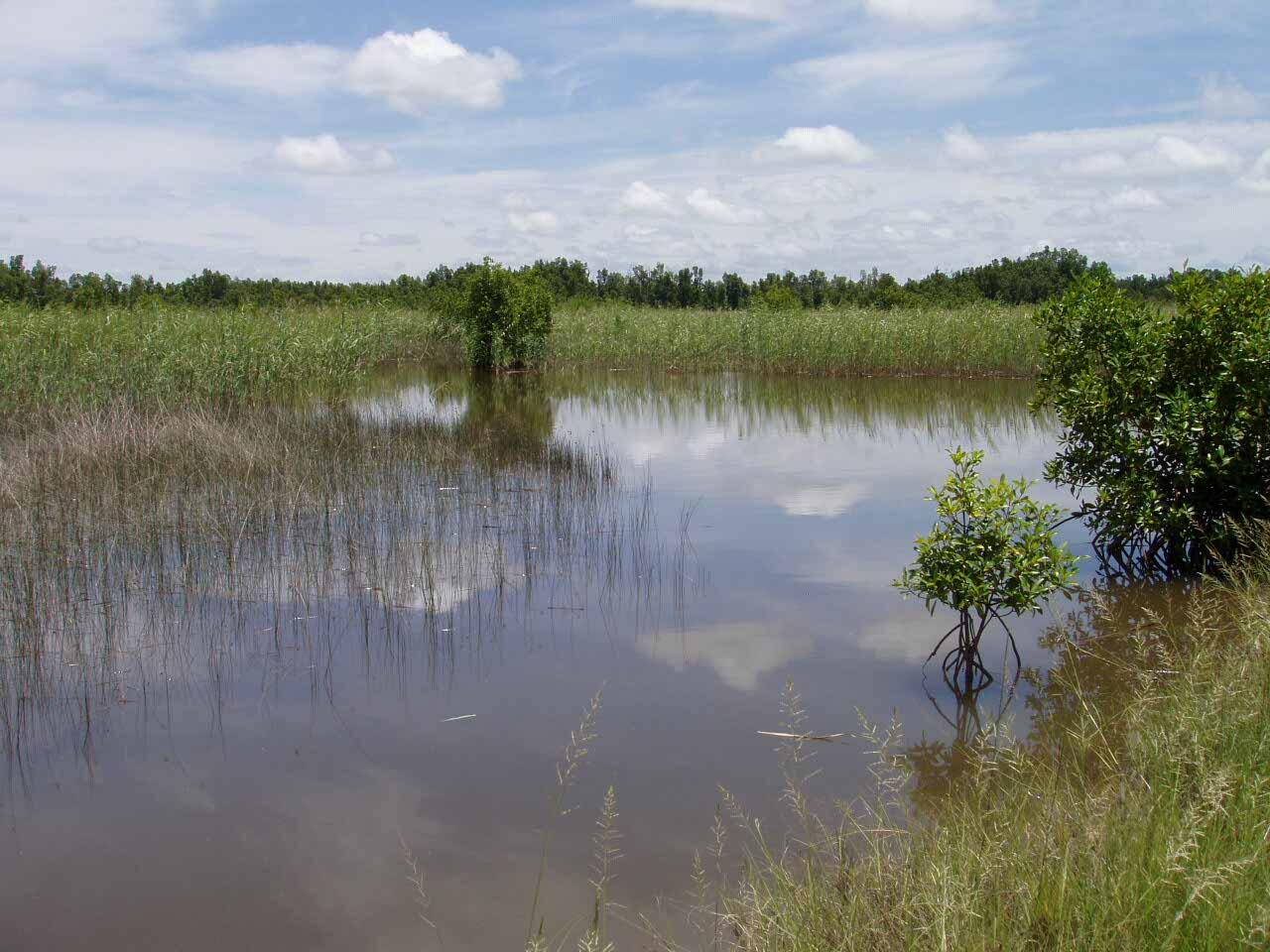
Areas of water **in floodplains** further away from the landward edge (not the first waterbody after entering the floodplains from the upland). These can be open floodwater bodies with deep water (sometimes dependent on high tides) but they can also be shallow with similar characteristics as the edge of floodwater, just that these sites are further away from the edge of the floodplains. These can also be sites where tall reeds characterise the land cover.

*3. Rice fields*

Flooded areas used to grow rice including rice nurseries.


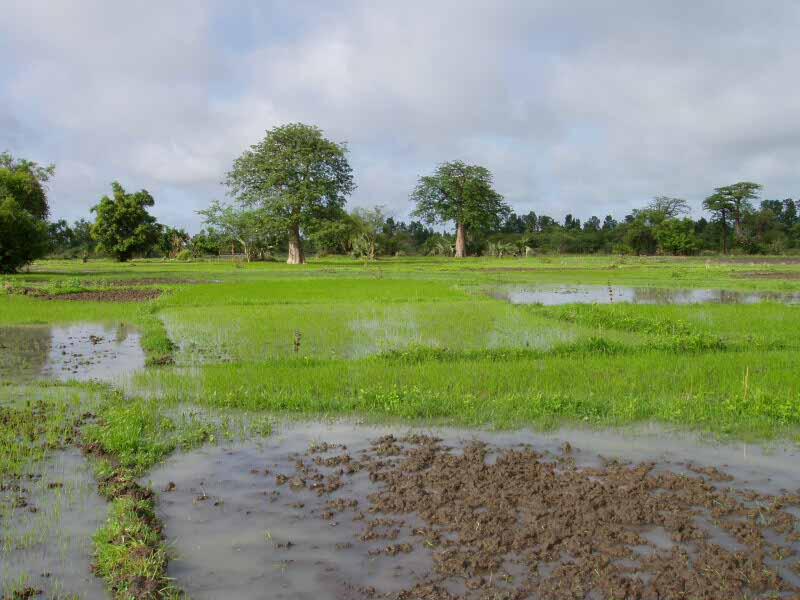
Rice fields can be found in floodplains or in upland, this should be indicated in the comments.

*4. Stream fringes*

Streams are water bodies with a more permanent character and are deep in the middle of stream. Mosquito habitats are usually associated with the fringes of these streams. Streams might be temporary but might also support puddles and pools on their fringes or in the middle of the stream bed when they dry up.


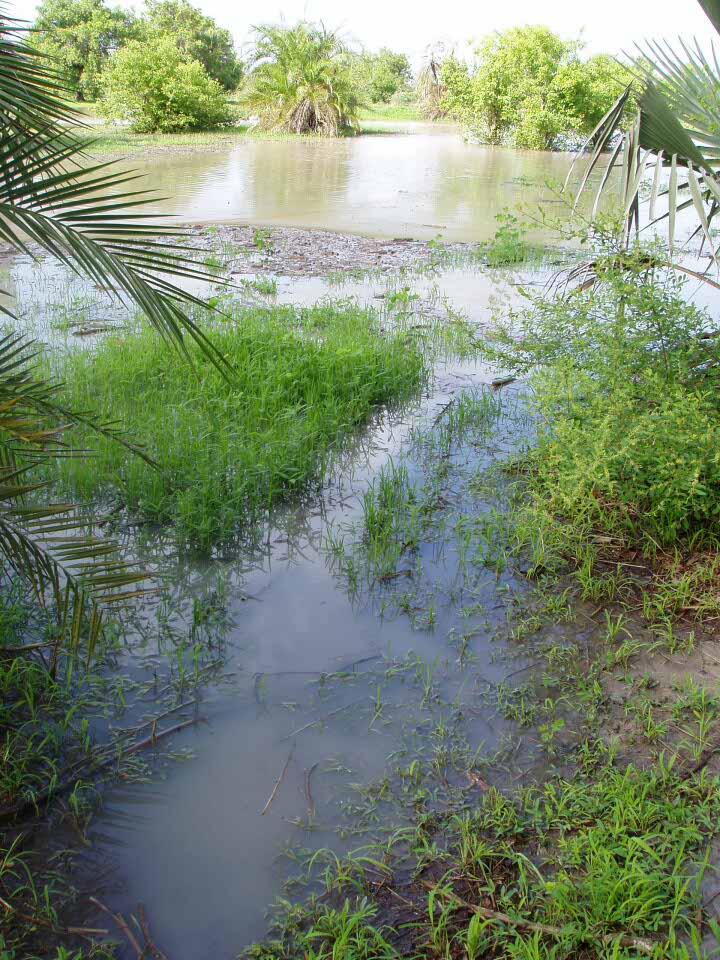


*5. Pools*

**Discrete** standing water bodies, medium-sized to large (greater than 2 m^2^), water originates from ground and rainwater and can be natural or man-made. Pools are relatively shallow and therefore not present throughout the year.

*6. Ponds*

Ponds are **discrete** water bodies of larger size and higher depths filled by groundwater. Ponds are permanent water bodies present throughout the year although their size may decrease in the dry season. The edges of ponds may serve as mosquito larval habitats.

*7. Footprints*

Footprints from people, cattle or other animals can form small holes in the wet ground where water can collect. This water can come from rain or from the ground, footprint areas are often found on edges of large water bodies (floodwater, pools and ponds).


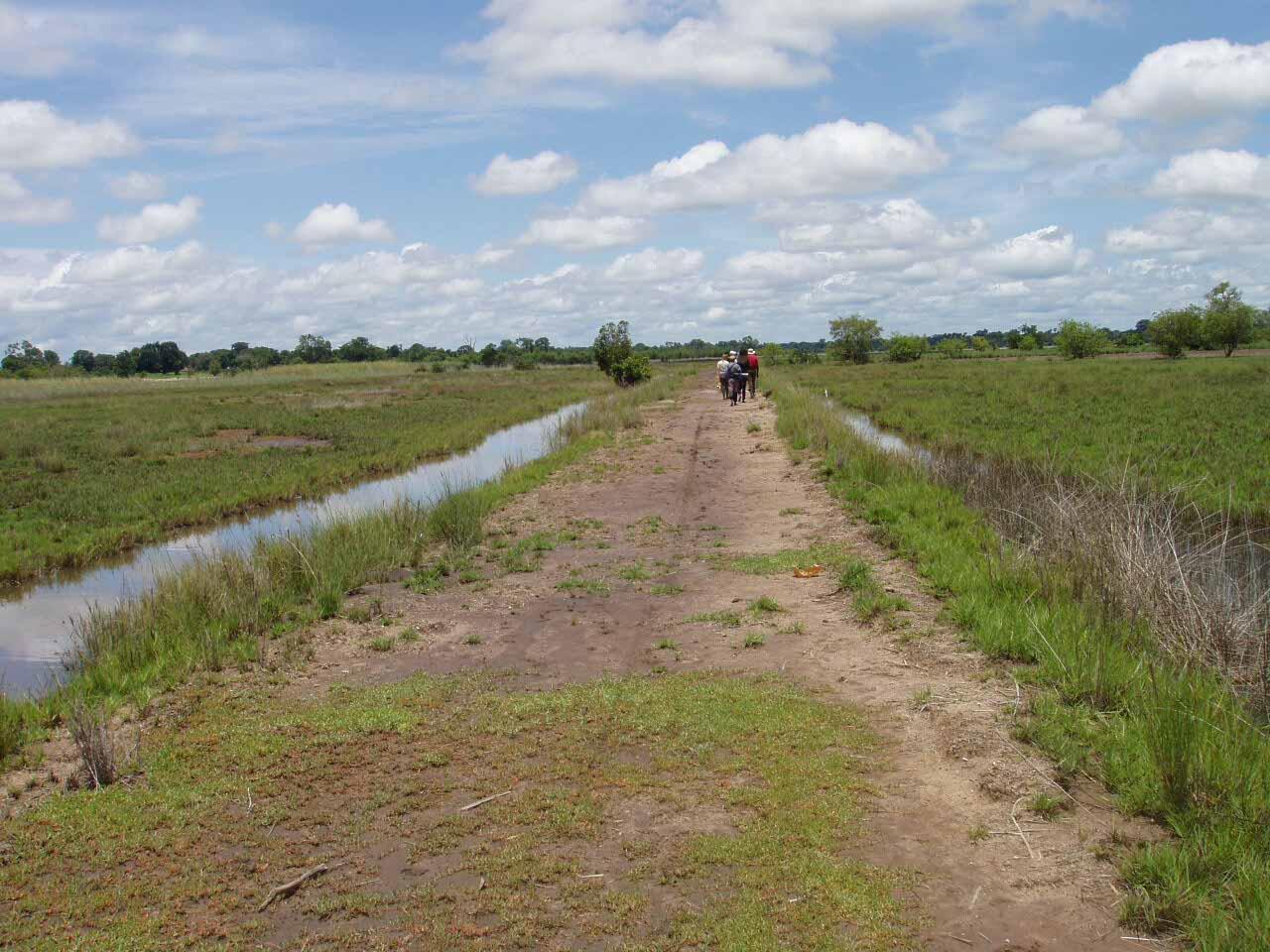
*8. Water channels*

Water channels are often man made. They may be used to channel water for example for irrigation associated with agriculture, or to drain the water (surface water run off). Channels are also often associated with road construction.

*9. Mangrove forest*

Habitat with dense mangroves near the edge of the river.

*10. Puddles and tyre-tracks*

Puddles are small to medium sized areas (less than 2 m across) where water stands on the ground after rain, they are always natural, filled by rain and water runoff.


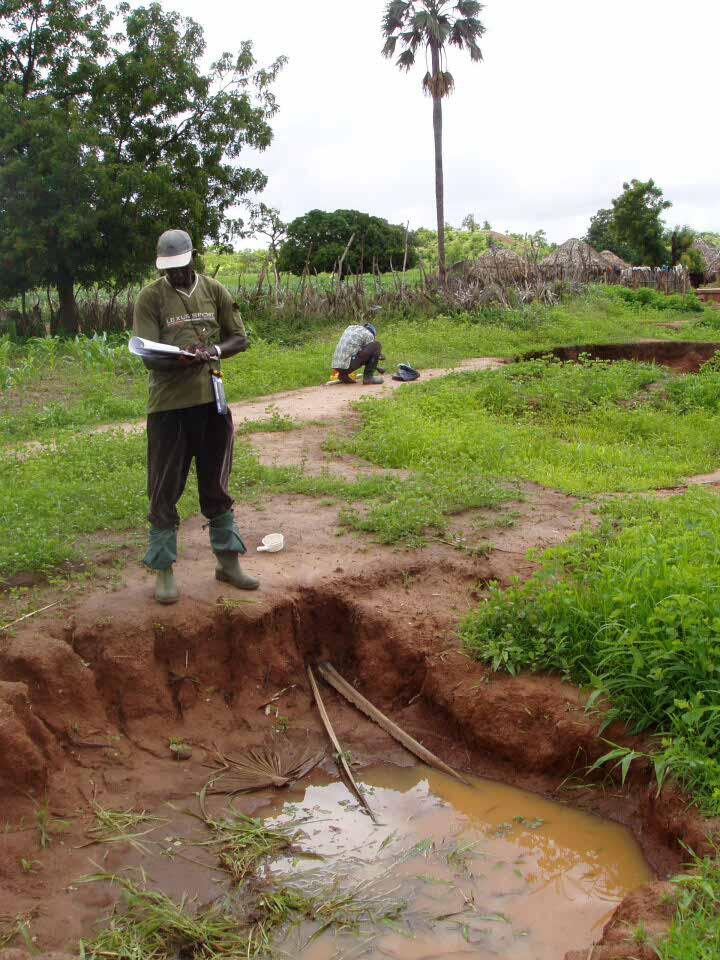
Tyre tracks are puddles that collect water due to marks left by vehicles in the ground that get filled with water after rains.

*11. Man-made constructions e.g. animal watering places near wells and market garden wells*

Man-made constructions that hold water either on purpose e.g. to water the livestock or by accident for example unfinished construction sites. These habitats are usually found in and close to villages.

Market garden wells (not the village wells) are small (2m in diameter), discrete deep holes filled with water, and can be found in agricultural fields and in floodplains and might represent important dry season refugia for Anopheles mosquitoes.

Indicate the type of habitat in the comments section of data sheet.

*12. Brick or sand pit*

Pits used to make bricks or dig sand.

**Operational mapping of potential mosquito breeding habitats**

- To find ALL potential mosquito breeding areas you have to observe your environment closely, you need to learn to know every m^2^ of your study area. It is your responsibility.
- Draw sketch maps of your study areas to help you familiarise yourself with your area
- You need to visit all areas with stagnant water that could be a potential breeding habitat for mosquitoes.
- Visit all areas. Seek consent from chiefs and communities (See Community sensitisation section) to visit the compounds to check for mosquito habitats. Inform the community properly about the work you are doing, answer all questions to the best of your knowledge and be friendly to ensure highest collaboration from the community.
- Ask people if they know any standing water around their compound and if they store water in any way. Always talk to people to get more information about possible breeding sites (stagnant water). Try to get as many community members involved in the monitoring of habitats as possible.
- Check all discrete water bodies that are less than 100 m in circumference by **dipping at least 10 times** in places that are likely to harbour larvae (edges, tufts of vegetation etc, see above). If the water body is large dip in regular intervals as you walk along the water body. It is important that the entire edge of the water body is sampled. In addition, inside the main (shallow) floodwater areas walk transects and sample. Always make several dips at one sampling site.
- Check fringe areas of large water bodies, mangroves and rivers in the area of your responsibility, check for man-made holes for irrigation, soil, stones and brick making, check the run off areas where water flows to the river, check the rocky areas, where water after rain can easily stand for some days or weeks, check open grassland and tyre tracks for any puddles from rain and any pools, check areas of agriculture where artificial ponds, irrigation channels and puddles and pools from irrigation can provide excellent breeding habitats for mosquitoes.
- Always search the sites closely, so that you can see the water surface and take water samples with a mosquito dipper. Try to go along or around the habitat since water might only be present in a small area of the habitat at the time of survey.
- Take at least 10 dips in any one area where mosquito larvae can be expected (edges of sites, around vegetation, shallow areas etc.).
- In your area within your cluster, you will be assigned a 5 km^2^ area to cover weekly.
- Record the position of any aquatic habitat you find and give the habitat a unique number (site ID) and GPS coordinates for quick reference in the field. The site ID will consist of the Cluster ID, and a continuous number starting with 001.
- Record only sites that contain water at the time of mapping. Any habitat that has been mapped containing water and given a site ID has to be revisited any time you re-map the zone even if it is dry, this you record in your data sheet. Any new site that you find containing water that has not been mapped before needs to be recorded and given a unique site ID that has not been given to another site before. Once a site has entered the mapping is has to be revisited every time you visit the area.
- The site ID is given to a habitat or environmental unit of habitats and needs to be different for each unit/habitat. Multiple puddles or small pools connected to one aquatic habitat will be represented by one site ID. Rice fields usually cover a larger area. These rice fields are represented by one site ID even if they are not totally flooded at the moment of mapping. You may decide that with more rain these rice fields will all be flooded and represent one aquatic habitat at times. If a mapped site/unit consists of several pools at the moment of mapping please indicate in comment section of the data sheet.

Combine habitats in one ID only if they represent an environmental unit, for example: several close puddles would be summarised by one site ID. Floodwater from the same river covered by grass and others covered by a different landcover type would be given a separate site ID, furthermore there could be a stream flowing through this environment but because of the difference in the larval habitat characteristics (stream fringe) this stream would be in its complete lengths summarised by another site ID. Ricefields related to that same stream would be represented by a separate site ID.

- GPS reading: record the GPS coordinates for each site ID, if the site is not only one discrete habitat but consists for example of a number of rice fields we need to record the extension of the habitat/unit. Take therefore at least one GPS readings at the beginning and one at the end of the site (and note these in the comments).
- Record in the provided data sheet the following:
- date of mapping
- your name
- cluster ID
- continuous site ID for habitat/unit
  landcover type (What **DOMINATES** the landcover in the area where you find the aquatic habitat? - Decide wherever possible for one landcover type only. If there is no single one dominating, but the land is equally covered by several vegetation types, then record multiple types in data sheet),
- Aquatic habitat types present at site ID (this can be multiple ticks at moment of mapping, a unit can for example consist of a pond and footprints at the edges),
- GPS coordinates (if large site measure extension by taking at least two coordinates),
- habitat size (<10m, 10-100m, >100m perimeter, if you are not sure walk along the site and count your steps, a large step is approximately 1 metre),
- water present or absent,
- water depth (shallow = less 50cm, deep = more 50cm, 50 cm is approximately the height of your knees),
- **number of *Anopheles* and Culicine early and late instar larvae and pupae.**

**Staff**

There are 15 Conventional LSM clusters (there are also 15 SIS LSM clusters) with an average extent of 5.5 km sq (‎Figure 2 and ‎Table 1). Each cluster will have one cluster leader and a minimum of two spray operatives. Some clusters may need more spray operatives due to their size or due to logistical constraints. Below is a table and map summarising the study clusters, their size and whether they are allocated as Conventional or SIS.


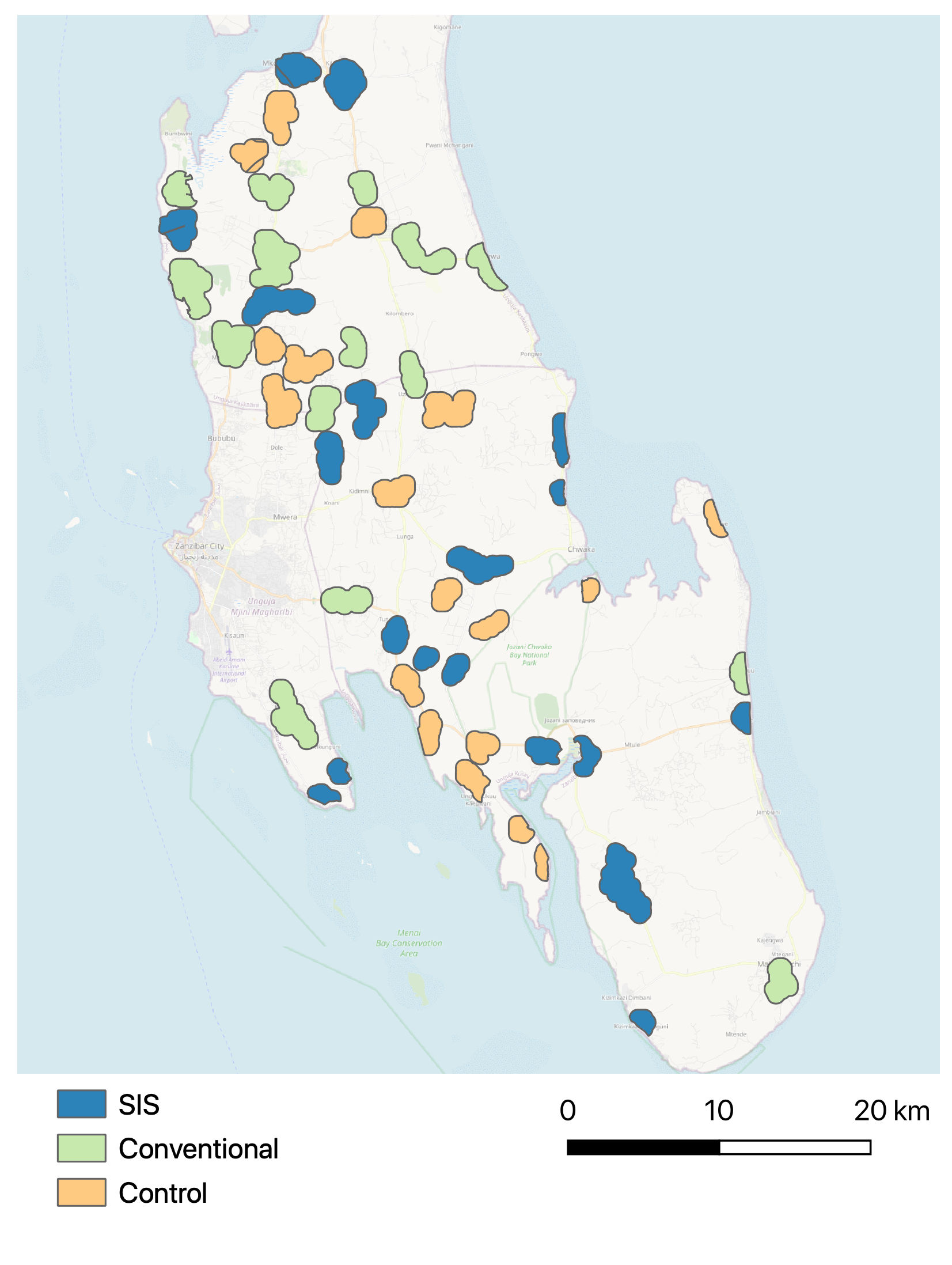


1. Map showing the location of the study clusters across Unguja Island, including those allocated as Conventional study clusters in green.
2. Summary of Conventional study clusters including area and study arm type.

| **Village** | **Area km sq** | **Arm type** |
| --- | --- | --- |
| Kisimani | 3.348 | Conventional |
| Ndagaa | 3.808 | Conventional |
| Bwejuu | 3.859 | Conventional |
| Tunduni | 4.144 | Conventional |
| Bumbwini | 4.249 | Conventional |
| Makunduchi | 4.767 | Conventional |
| Tunguu | 5.157 | Conventional |
| Donge Mbiji | 5.194 | Conventional |
| Kiwengwa | 5.207 | Conventional |
| Miwani | 5.39 | Conventional |
| Mfenesini | 6.586 | Conventional |
| Upenja | 7.178 | Conventional |
| Fujoni | 8.087 | Conventional |
| Mchamba Wima | 8.226 | Conventional |
| Mahonda | 9.074 | Conventional |

**Roles and responsibilities**

**Cluster leader**: Is responsible for ensuring the mapping, larviciding implementation and monitoring is done according to this SOP. S/He ensures all logistics are in place for the success of the program. In addition, the cluster leader has the responsibility to conduct spotchecks to assess the performance of the team. S/He responsible for collecting data forms from the team and handing them over to ZAMEP.

**CHECK LIST of Field Equipment for HABITAT MAPPING**

- 1 backpack
- 1 GPS and spare batteries in plastic bag (or tablet)
- 1 Standard Dipper with handles
- 1 small dipper
- 2 plastic pipette
- 1 map
- 1 copies of habitats definition
- data recording forms in plastic holder or tablet
- 4 sheets of plain paper
- 1 clip board
- 2 pencils
- enough drinking water
- hat, rain coat, boots

**Protocol for Larviciding with *Methoprene* in Zanzibar**

Silas Majambere, Prosper Chaki, Andy Hardy

Note: There are three forms of Larval Source Management (LSM): habitat modification, habitat manipulation and larviciding. This protocol is detailed for larviciding, but wherever possible habitat modification or manipulation (also termed as environmental management) should be the priority.

**Goal:** To demonstrate that a combination of a state-of-the-art remote sensing (satellite and drone-based), coupled with an innovative use of smartphone technology (Zzapp Malaria), will offer a more efficient and cost-effective approach to Larval Source Management (LSM) compared to conventional larviciding for eliminating diseases like malaria.

**Specific Objectives of the Larviciding intervention**

- To manually map water bodies in the “Conventional larviciding clusters” (“CL”). This procedure is described in the Supplementary Document “SOP_Mapping_Conventional”
- To map water bodies in “SIS clusters” (“SIS”) using drone technology. This procedure is described in the Supplementary Document “Mapping Quality Study”
- To design and implement larviciding interventions with Methoprene in “SIS” and “CL” clusters
- To measure the cost and impact of the SIS on mosquito density and malaria case-incidence.

**Study Hypothesis**

This project is a proof of concept study that aims to demonstrate that the Spatial Intelligent System (SIS) can achieve efficient and cost effective larviciding to control malaria in an operational pre- elimination setting in Zanzibar.

**Evaluation of Larviciding Success**

In our study we hypothesize that in comparison to the non-intervention clusters, the clusters receiving CL and SIS will have a reduced number of mosquitoes and a reduced malaria case incidence.

**Success depends on:**

- Identification of ALL available aquatic habitats within the study area
- Treatment of ALL aquatic habitats in required dosages
- Proper performance of the larvicides
- Treatment in regular intervals so that no late instar larvae can ever be recorded in the sites (every three weeks)
- No adult emergence takes place in any sites due to sufficient larval control.

**Timeline**

- Collection of baseline data from March-June 2021
  - Availability of aquatic habitats
  - Colonisation of habitats with Anopheles mosquitoes
  - Adult densities in houses
- Recruitment of spray operatives from intervention communities
- Training on mapping water bodies, larval sampling and application of larvicides in February
- Implementation of 3-weekly larviciding in intervention sites from June 2021 to October 2021
- Monitoring and Evaluation of intervention from June 2021 to October 2021
  - Colonisation of habitats with Anopheles larvae (weekly)
  - Adult mosquito densities in houses (at sentinel sites)
  - Malaria incidence in population (MCN, MEEDS case data capture programs)

**Study site**

The study will be conducted in Unguja, in 45 clusters with 15 clusters as control, 15 for conventional larviciding and 15 for SIS.

**Methoprene larvicide**

The insect growth regulator product Altosid XR-G was used which includes the active ingredient Methoprene (1.5%). Altosid XR-G releases effective levels of the Altosid insect growth regulator for up to 21 days after application. Treated larvae continue to develop normally to the pupal stage where they die. Altosid insect growth regulator has no effect on mosquitoes which have reached the pupal or adult stage prior to treatment.

Application rates for *Aedes, Anopheles,* and *Psorophora* spp.: Altosid XR-G at 5.6-11.2 kg/ha.

Application rates for *Culex, Culiseta, Coquillettidia*, and *Mansonia* spp.: Altosid XR-G at 11.2-22.4 kg/ha. Within these ranges, use lower rates when water is shallow [< 60 cm] and vegetation and/or pollution are minimal. Use higher rates when water is deep [> 60 cm] and vegetation and/or pollution are heavy.

**Application Equipment**

- **Hand application:** Granular formulations (CG) may be applied by spreading by hand, similar to scattering seeds by hand. However, it is difficult to obtain an even application or maintain the recommended application rate. It is very important for the field staff to practice this exercise well to gain experience following the guidelines from the calibration workshop. For hand application of granular formulation we will use a bucket on a comfortable carrying strap to be hung around the shoulders allowing the bucket to rest on the belly, the carrying strap can be adjusted to individual comfort.
- **Motorized spreaders:** Motorized backpack spreaders may also be used for the application of granular formulations of larvicides. These use an air blast to spread granules and might therefore be considered when larger distances need to be covered and are not accessible otherwise. These applicators are fairly heavy, a significant consideration while walking through mosquito larval habitats. Therefore, this equipment might only be used when no other option is available. The granule spreaders need to be calibrated taking into account the amount of larvicides (discharge rate) and the walking speed to ensure an even distribution of the larvicide in the right quantities. These spreaders need to be well maintained according to manufacturer’s recommendations. In both SIS and Conventional clusters the TGS30 TOMAHAWK 4 Gallon Backpack Motorized Spreader for Granular Insecticides was used.

**When to use what?**

Granule application by hand:

- Effective in sites with open water bodies, but also in sites with emergent or floating vegetation
- Granules penetrate vegetation and drop on water surface

Motorized back-pack spreaders:

- Effective in sites with open water bodies, but also in sites with emergent or floating vegetation
- Granules penetrate vegetation and drop on water surface
- Granules can often be spread for a larger distance than hand applied granules and can therefore be used to treat less accessible sites

**Recruitment of staff**

The spray teams will be recruited from the communities in clusters selected for the intervention. These will have to be fit people willing to work in tough conditions and taking pride from their work. Each intervention cluster will have a supervisor who will ensure recruitment and training of the spray team in their cluster, with the support of ZAMEP.

**Training and posting of spray operatives**

All spray operatives will be trained in their respective intervention areas in February 2021. The main objectives of this training will be to give them a first hand knowledge about mosquito larvae and to show them that mosquitoes can breed in all sorts of water bodies and this should help them understand that they should **NOT miss any water body during routine spraying**. In the conventional larviciding clusters, spray operatives will be allocated well defined areas that they will have to know very well and be responsible of during the entire spraying campaign. These areas will be flagged using sticks with a red or white rag on top to allow spray operatives to locate them easily. During the first days of training, the spray operatives will learn that all aquatic habitats have to be treated regardless whether they contain larvae or not. They also need to understand that they should **treat ALL water bodies every three weeks**.

Spray operatives will have to be aware that the water bodies will expand and new ones occur during the rainy periods and by that time they should have gained experience with spraying. That experience should help them cope with the rainy season changes and be able to still cover the area every three weeks. Follow up of spray operatives and on job training will be done by the cluster supervisor who will assess the performance of every spray operative regularly. ZAMEP will help in training and follow up.

**Storage and Distribution of Larvicides**

Larvicides will be supplied from ZAMEP and a data sheet of monthly stock release will be signed between the cluster managers and the project manager at ZAMEP.

Before taking the larvicides to the different clusters, enough larvicides for the month will be transferred into bags and this stock of larvicides will have to be stored in a secure place, easy to reach by the spray team. This could be at the health post or any other public office in the area, to be agreed by the local community. This place should be a tight place with no leakage of water and preferably lockable. The cluster supervisor should be the only person to access that area wherever possible to avoid misuse of the larvicides.

Every spraying day the cluster supervisor will take the necessary amount of larvicide needed for that day together with data sheet and will give each team the necessary amount of larvicide for that day. The spray operative should always have more than they need to spray daily to avoid circumstances where they would run out of larvicides when the cluster supervisor is not in close vicinity. The larvicides not used in the field will be handed over back to the cluster supervisor who will collect the leftovers from buckets or spreaders in the original bag weigh it and record in the data sheet. This bag should be used first the following spraying day before starting new ones. All the remaining larvicides will be taken back to the store in the area.

The cluster supervisor will record the amount of larvicide given to each team of spray operatives in a data sheet (see **Data sheet 1**) that he will return to the project manager at the end of the month. The cluster supervisor will spend around 15 minutes with spray operatives discussing activities of the day and after distributing the larvicides and sending out all the spray operatives, he/she will start their spot checks. All spray operatives should have started their work latest at 7:30 am. At the end of the day, the cluster supervisor will meet the spray operatives again to discuss the work of the day and record in the data sheet the amount of larvicide returned that day. If there are issues to address such as sites that were not covered, problems with the equipment or any other problem that could occur they should be addressed straight away and a solution found. If there are sites that could not be treated, an immediate solution has to be found in order to treat all sites every three weeks. If these are issues, the cluster supervisor and spray operatives cannot solve alone, the cluster supervisor will immediately report them to the project manager who will take appropriate action.

**Implementation of operational larviciding**

- Early morning, the cluster supervisor will take the necessary amount of larvicides for a day and proceed to meet his team.
- Fieldwork will start as early as possible in the morning preferably just after sunrise to be able to complete work before the hottest temperatures of the day.
- The cluster supervisor will distribute the larvicide to the spray operatives in necessary amounts and record this in his data sheet and send the teams in their allocated areas. Spray operatives should have enough red rags that they will use to landmark their area for easy remembrance.
- Message for spray operatives:
  - For the first day of spraying stick a flag where you have started.
  - If you are spraying a large swath (i.e. ricefields), ensure at least half a meter overlap with the next sprayer.
  - Once you start spraying, try as much as possible to walk with the same pace of approximately 60 m/min (determined during calibration). This will allow you to spray the water surface evenly and treat all water bodies with the appropriate amount of larvicide.
  - If you reach a water body where accessibility is very difficult, make sure you treat the edges of that water body and project the larvicide as far as it can get.
  - At the end of the day, put a red flag where you have stopped and start there the following day. Continue this motion and spray all water bodies in your area, until you reach the limit of your area. You should see the red flags that show the delimitations of your area before you are satisfied you have covered the whole area assigned to you.
  - At the end of the day, hand back all larvicides that have not been used in the field that day and discuss your day with the cluster supervisor
  - Remember that ALL water bodies in your area have to be sprayed every 3 weeks.
- After sending off their team, the cluster supervisor will start supervision rounds.

**Larval Density Surveys**

Larval density will be recorded weekly in the same two sentinel sites in each cluster as during the baseline data collection both in intervention and non-intervention clusters following the same standard procedures as during the baseline data collection. The cluster manager will be responsible for the larval collections.

Data on larval density will be recorded in the same data sheet as for the “Larval density in sentinel sites data sheet” used for the baseline data.

**Monitoring and evaluation of success**

**Adult Surveys**

Adult surveys for monitoring and evaluation of success of intervention and reduction in transmission will be implemented using sentinel sites routinely collected by ZAMEP.

**Community sensitization**

It is mandatory to inform and gain consent from the administration, community leaders and the community members before any larviciding can take place in the intervention areas. Community members are usually very concerned about any pesticide applied by research teams. There is usually the fear that pesticides applied on water could affect human beings or live stock.

**Chiefs in the area where spraying will take place should be visited and informed about the planned activities, and they would be asked to help organise meetings with their communities**. Community leaders need to be informed and with their help community meetings need to be held. Any questions and concerns of the community need to be answered to the best of your knowledge. Questions that cannot be answered immediately need to be discussed with the scientists and information brought back to the community. Everyone involved in the sensitisation process should have the same message to the population. Therefore, a leaflet and a frequently asked questions fact sheet (see below) will be distributed during the sensitization meetings to those chairing the meeting and anyone else in the community who can read and is interested.

**The key messages to spread during the sensitization:**

- Malaria is transmitted by one type of mosquito, Anopheline mosquitoes (show adult mosquitoes). These mosquitoes breed in water and while in water they look like small worms (show larvae);
- We want to find all the water bodies around this area, in the swamps, ricefields and around your compounds and see whether they contain mosquito larvae or not;
- Because mosquitoes can breed in any type of water including ricefields, we want to survey the ricefields, but we will make sure not to disturb any crops;
- We have larvicides that only kill mosquitoes in the water.
- These larvicides are absolutely safe, they are not harmful to humans, to cattle, or fish and they don’t affect crops production;
- These larvicides are very efficient, they have been used in different parts of the world. We know that they kill mosquito larvae and we want to see whether we can use that tool for malaria control. By killing larvae we will reduce the number of adult mosquitoes entering your houses and hopefully reduce malaria exposure and incidence;
- You will see people from your villages and ZAMEP in the swamps and around your compounds. They will be carrying spraying equipment like motorised spreaders, and buckets (show colour photographs of people using this equipment);
- Because mosquitoes occur in ricefields, we would like to have your permission to walk along the edges of your rice plots and spray there. We will try as much as possible not to disturb your crops. Wherever you feel the spray operatives have mistakenly disturbed your crops, approach the field team leader, and he will endeavour to find an appropriate solution to the matter.
- We would like you to interact with the spray teams, talk to them about anything unusual you notice, including whether the number of mosquitoes in your houses has reduce or not.

Daily Timetable for Spray operatives

- 6:30-7:00: Get the equipment ready (backpack, flags, cup…)
- 7:00-7:30: Meet with cluster manager, get the larvicide for the day, data forms
- 7:30-13:30: Routine spraying and monitoring
- 13:30-14:00: Clean equipment; report to cluster manager and hand over larvicide leftovers

Daily Timetable for Cluster Manager

- 6:00-6:30: Get the larvicide from local store, get the data sheets ready
- 6:30-8:00: Meet with spray operatives in central areas; distribute enough larvicide for the day
- 8:00-12:00: Select randomly where to supervise larviciding operations
- 12:30-14:00: Meet spray operatives, collect larvicide leftovers and data forms

**Mapping Quality Study**

**Introduction**

This study presents a framework for the operational deployment of drone and smartphone technology to collect and deliver spatial intelligence for use in Larval Source Management (LSM) programmes.

A growing number of studies have demonstrated the use of drone technology for mapping *anopheles* mosquito breeding sites (Fornace et al. 2014; Hardy et al. 2017; Stanton et al. 2021; Carrasco-Escobar et al. 2019; Schenkel et al. 2020; Hardy, Oakes, et al. 2022) yet, so far, there have been no reported attempts to benchmark the success of this approach against conventional, ground-based mapping of mosquito breeding sites. As such, a key question remains: does drone technology provide improvements over ground-based mapping efforts?

The aim of this study is to quantify and compare the accuracy of drone-based and conventional-based mapping of potential mosquito breeding sites, benchmarked against a ground-truth survey. These findings can then be used to calculate unit cost per potential breeding site mapped and the cost per *anopheles* positive breeding site mapped, described later in this document (page 18-19)

**Methods**

*Study site*

The mapping quality study carried out over eight sites across the island of Unguja, Zanzibar (‎Figure 1). Each site measured 600 x 600 m, this being large enough to evaluate the scalability of the mapping approaches over variable land cover types whilst being small enough to be covered in one day by a field team to make observations for validation purposes.

The sites were randomly located within the 15 conventional arm study clusters – the design of these clusters for the wider study are described in the main manuscript.

The sites represent a range of land cover types and a range of hydrological conditions, i.e. some sites with large scale irrigated/rainfall fed rice paddies; some sites dominated by dense forest with few surface water bodies; some sites dominated by dwellings with isolated water sources; sites over high infiltrating soils with very little surface ponding.


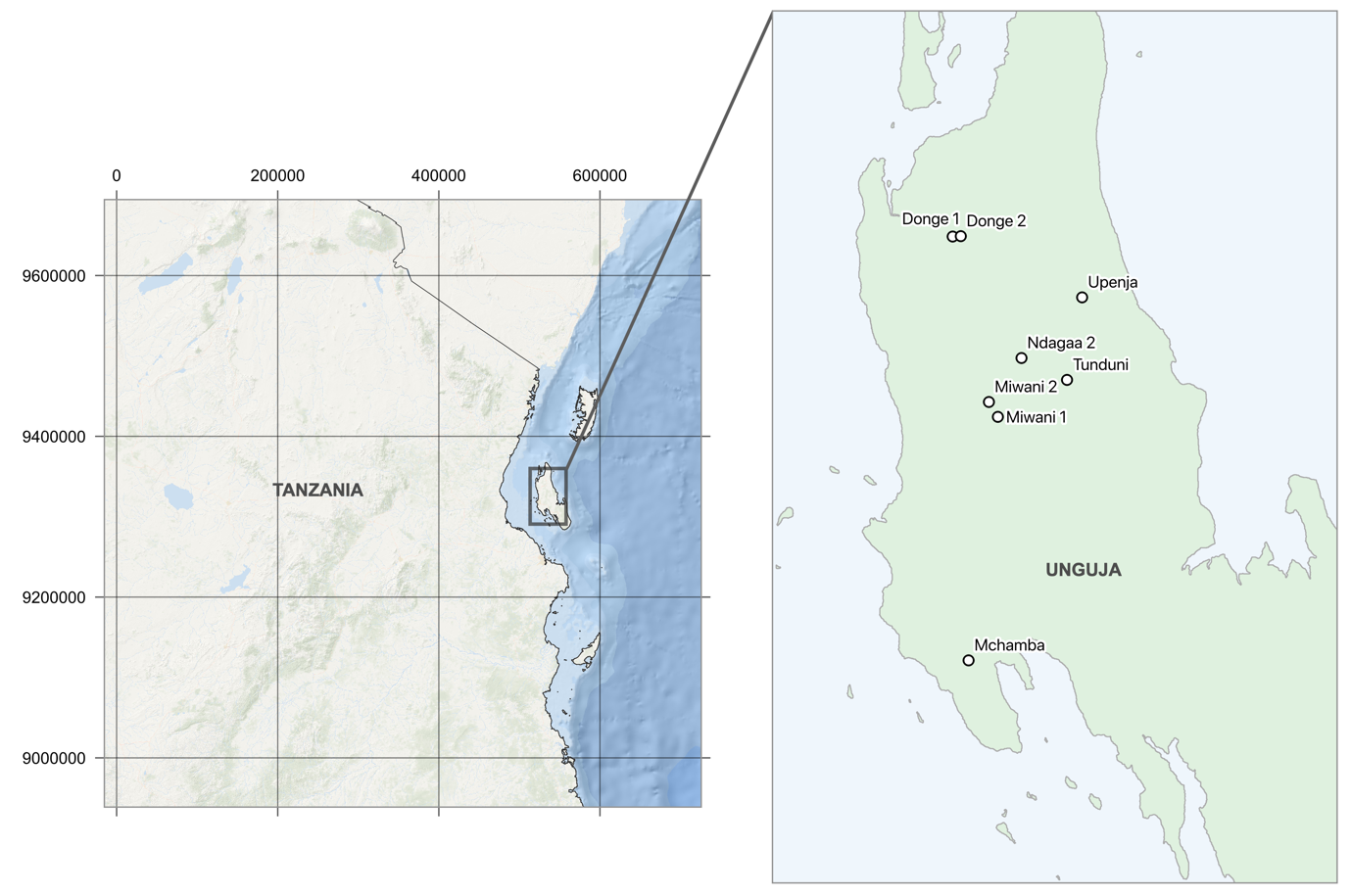


1. Location of mapping quality sites where coincidental drone mapping, conventional mapping and ground-truth mapping took place.

*Ground-truth data collection*

Each of the eight mapping quality sites were split into a series of 10 x 10 m grid squares (‎Figure 2). Using a modified version of Zzapp Malaria, fieldworkers visited as many grid squares as possible, recording any surface water feature they encountered together with the characteristics of the water body and larval survey. The assumption is that a fieldworker is able to identify any surface water within 10 m of where they are standing. A 10 x 10 m grid square is recorded as a Treatment Unit (TU) where it is fully or partially covered water (no matter how small), i.e. an area that a field larviciding operative would need to visit and treat.

Larval surveys at each grid square with water in it was carried out using a purposive dipping strategy using a 350 ml dipper, whereby a minimum of ten dips were made in places most likely to harbour larvae, such as around clumps of vegetation or protruding substrate, amidst floating debris, and along the periphery of the water body (Hardy et al. 2013).

At each site there we were 3,600 grid squares. Field teams attempted to visit every grid square but this was not always possible due to difficulties in access particularly in areas with dense thicket or extensive flooding.


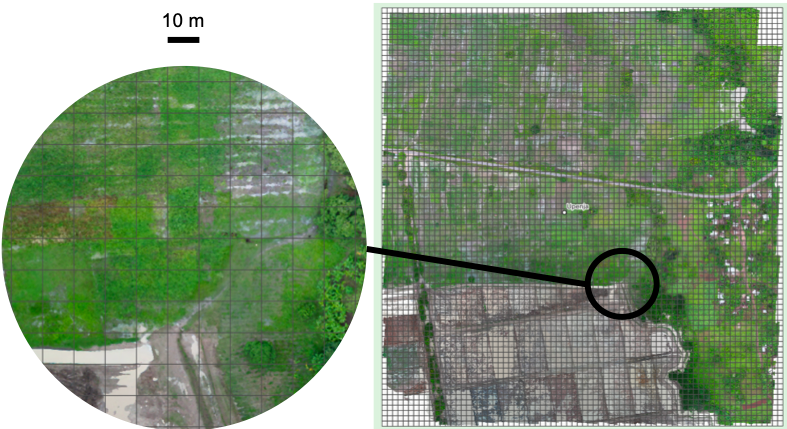


1. Example mapping quality site, split into a series of 10 x 10 m grid squares.

*Drone-based mapping*

In March 2019 eight members of ZAMEP were trained by Aberystwyth University and the Tanzanian Flying Labs in the use of rotary quadcopter drones. This included i) flight planning, ii) manual and automatic flying, iii) processing imagery and iv) mapping water sources in drone imagery using Geographical Information System software (as described below).

Drone surveys were carried out in May 2021 in line with Tanzanian Civil Aviation Authority regulations. Prior to drone deployment, community engagement was carried out to inform and gain permission from local communities and reported in Hardy et al. (Hardy, Proctor, et al. 2022). Aerial photos were captured using a DJI Phantom 4 Advanced+, weighing ~1.3 kg, with a maximum flying time of 30 minutes, fitted with a standard 1-inch CMOS red, green, blue camera with an effective pixel count of 20 M. The flights were planned and executed using DroneDeploy mobile application (DroneDeploy 2021). Flight parameters were designed to maximize the coverage that could be made with each flight: Flying height ~120-150 m, flying speed set to 15m/s, images at 75% Front Overlap, 65% side Overlap.

Agisoft MetaShape’s Structure from Motion (SfM) routine was used to construct orthomosaics for each site with a pixel resolution of 20 cm. This provided a compromise on computational burden (data processing and storage) whilst maintain sufficient resolution to capture surface water body features that have the potential to be mosquito larval habitats. Details of the SfM processing parameters can be found in Hardy et al. (2022). A MacBook Pro (Intel core i7, 16GB RAM, HD Graphics 1536 MB) was used for image pre-processing and subsequent analysis as described below.

Technology Assisted Digitising (TAD) was used to map surface water features. Manual digitizing has the advantage that the human operator is able to consider the context of a feature and its likelihood of belonging to a particular thematic class (i.e. surface water). But this approach is time-consuming. The TAD approach employs a region growing tool (a freely available plugin for QGIS available at: https://github.com/gro5-AberUni/RegionGrow), developed for this project and reported in Hardy et al. (2022), that significantly speeds up the digitizing process whilst maintaining a high degree of accuracy and precision. The TAD approach was shown to provide significant improvement over a machine learning-based supervised image classification.

*Conventional mapping*

The protocol for conventional mapping was defined by experienced LSM practitioners from the Pan-African Mosquito Control Association (PAMCA). A detailed description of this protocol can be found on pages 1-8 in this document. In Zanzibar, previous LSM initiatives have made use of community volunteers for mapping and treating potential breeding sites. Community members were recruited through consultation with Shehia committees (small administrative unit) and trained how to produce baseline breeding site maps. This involves 1) a preliminary paper-based sketch of the target area, splitting the area into chunks, 2) walking each chunk and identifying potential breeding sites, 3) paper-based record of breeding site including observations of larvae/pupae and GPS location.

Conventional mapping was carried out within the 15 Conventional Arm study clusters. As described above, the eight mapping quality study sites were located within these areas enabling a direct comparison of the conventional and drone-based mapping approaches, alongside a coincidental ground-truth survey. The paper-based data collection approach used in conventional mapping was not specifically designed for the mapping quality study. As such, there were instances where a single GPS point was recorded at the edge of a more extensive potential breeding sites, such as an inundated rice paddy. In these instances, every 10 x 10 m grid square covering the rice paddy would be recorded as a positively identified TU.

*Analysis*

For each site, the percentage of TUs and TUs testing positive for anopheline larvae correctly identified by the SIS and Conventional mapping approaches was calculated, giving a measure of mapping quality bench-marked against the ground-truth survey. Mean percentage of correctly identified TUs (and *anopheles*-positive TUs) was then calculated across the eight sites to summarise the quality of the two mapping approaches. A paired T-test was to compare the two mapping approaches with the null hypothesis that there is no significant difference in the quality of the two mapping approaches.

**Bibliography**

Carrasco-Escobar, G., Manrique, E., Ruiz-Cabrejos, J., et al. 2019. High-accuracy detection of malaria vector larval habitats using drone-based multispectral imagery. *PLoS Neglected Tropical Diseases* 13(1), p. e0007105.

DroneDeploy 2021. DroneDeploy [Online]. Available at: https://www.dronedeploy.com/ [Accessed: 15 October 2021].

Fornace, K.M., Drakeley, C.J., William, T., Espino, F. and Cox, J. 2014. Mapping infectious disease landscapes: unmanned aerial vehicles and epidemiology. *Trends in Parasitology* 30(11), pp. 514–519.

Hardy, A., Makame, M., Cross, D., Majambere, S. and Msellem, M. 2017. Using low-cost drones to map malaria vector habitats. *Parasites & vectors* 10(1), p. 29.

Hardy, A., Oakes, G., Hassan, J. and Yussuf, Y. 2022. Improved Use of Drone Imagery for Malaria Vector Control through Technology-Assisted Digitizing (TAD). *Remote sensing* 14(2), p. 317.

Hardy, A., Proctor, M., MacCallum, C., et al. 2022. Conditional trust: Community perceptions of drone use in malaria control in Zanzibar. *Technology in society* 68, p. 101895.

Hardy, A.J., Gamarra, J.G.P., Cross, D.E., et al. 2013. Habitat hydrology and geomorphology control the distribution of malaria vector larvae in rural Africa. *Plos One* 8(12), p. e81931.

Schenkel, J., Taele, P., Goldberg, D., Horney, J. and Hammond, T. 2020. Identifying potential mosquito breeding grounds: assessing the efficiency of UAV technology in public health. *Robotics* 9(4), p. 91.

Stanton, M.C., Kalonde, P., Zembere, K., Hoek Spaans, R. and Jones, C.M. 2021. The application of drones for mosquito larval habitat identification in rural environments: a practical approach for malaria control? *Malaria Journal* 20(1), p. 244.

**Costing Methods**

The costing took the societal perspective in that it included both provider (health system) and community (beneficiary) resources consumed. The cost of each mapping and intervention approach (SIS and Conventional) was determined using activity-based costing and the ingredients approach using a mix of top-down (reviewing and allocating centrally held data e.g. on personnel and equipment costs and using a suitable proxy to allocate costs to specific activities, geographical areas or study arms) and bottom-up (micro costing of specific activities) methods.

Activities (and sub-activities) for each mapping approach were identified, defined and categorised as either start-up (only done in the start-up phase of the project) or regular (would need to be repeated regularly during implementation). Activities were also categorised as either research (conducted solely for the purpose of research, i.e., the ground truth survey) or implementation (those that would be needed under routine implementation). Coding of activities as start-up or regular and research or implementation was done independently by authors (EW and AH) with a discussion to reconcile any discrepancies.

A set of pre-defined cost categories (personnel, equipment, consumables, transport, other) and sub-categories was used to systematically identify, and record resources used in each activity through direct observation, financial project record review and via discussion with ZAMEP staff. Donated resources (items for which no financial resources changed hands, e.g. meeting rooms and community member time) were also identified and recorded.

The quantity and unit price of each resource used was recorded, or for donated items it was imputed using market rates or minimum wage. Resources were categorised as capital if they had a useful life of more than one year and a unit cost of greater than US$100, otherwise they were defined as non-capital. Each cost item was coded as ‘SIS’ (used exclusively in the SIS study arm), ‘Conventional’ (used exclusively in the conventional study arm) or ‘both’. Costs which were categorised as ‘both’ were split equally between arms.

Costs were collected in the purchase currency, and converted to US$ using exchange rates at the midpoint of 2021 (18/06/21). Observational data was collected by colleagues in Zanzibar with remote support from the Liverpool team (COVID-19 prevented us from conducting on-site data collection together as planned).

Cost data was analysed according to standard methods i.e. capital items were annualised by dividing by their useful life (financial cost analysis) and by dividing by the useful life and applying a 5% discount rate (economic cost analysis). Research costs were removed from the analysis since these are deemed irrelevant for operational cost comparison purposes. All cost analysis was done in Microsoft Excel using custom designed costing spreadsheet. Costs are reported as annualised costs, in financial and economic terms (‎Table 1). For ease of reading, we will describe economic costs in the text, but tables show financial and economic costs.

1. Difference and suggested uses for annualised, unannualised, economic and financial cost data

|  | Unannualised cost | Annualised cost (Adjusted) | Uses |
| --- | --- | --- | --- |
| Economic cost | Value of donated items included  No annualisation | Value of donated items included  Annualisation over the useful life with discounting  (Adjusted averages start up activities over the useful life) | Gold standard representation of resources consumed for use in comparison with other economic studies |
| Financial cost | Value of donated items not included  No annualisation | Value of donated items not included  Simple annualisation over the useful life  (Adjusted averages start up activities over the useful life) | Best indication of financial cost for use in budgeting |
| Uses | Best indication of up-front costs. Useful for preparing financial budgets | Best indication of average costs over time, especially if adjusted and useful for comparing with programmes of different length |  |

The total cost of mapping and associated activities was calculated for the SIS and Conventional study arm firstly using the crude (unadjusted) annualised costs, and secondly by adjusting the annualised costs such that the cost of start-up activities was averaged over their useful life (‎Table 1). For example, communication and sensitisation materials developed were assumed to last five-years before they would need to be updated, hence only 1/5^th^ of these costs would be included in the adjusted annualised costs to reflect the share of this resource ‘consumed’ in an average year.

The following unit costs for SIS and Conventional arms could then be calculated:

Unit cost 1: Cost per waterbody (TU) correctly identified and mapped (number and m^2^ area of water)

Unit cost 2: Cost per anopheline habitat (water body with anopheline mosquito larvae in it) correctly identified (number and m^2^ of water)

Unit cost 3: Cost per breeding site treated (number and m^2^ area of water)

Unit cost 1 (per waterbody) and 2 (anopheline habitat correctly identified) were calculated using data from the Mapping Quality study. Since this study was conducted in a smaller geographical area than the SIS and conventional mapping was conducted and costed, we adjusted the estimated TU and habitats found in the GTS upwards, using the proportion GTS area mapped relative to the SIS or Conventional area mapped (see Table #).

**Supplementary Cost Data**

**Table 1 Input variables used in cost-effectiveness simulation modelling**

| **Arm** | **Category** | **Variable** | **Base case** | **Worst case◊** | **Best case◊** | **Distribution function** | **Base case** | **Worst case*** | **Best Case** |
| --- | --- | --- | --- | --- | --- | --- | --- | --- | --- |
| SIS | Costs (Economic):Startup | Community sensitisation | 4,852.81 | As base case | 3,396.97 | Point estimate | Measured cost from study | Base case | Base case |
|  |  | Training | 1,422.74 | As base case | 3,396.97 | Point estimate | Measured cost from study | Base case | Base case |
|  |  | Mapping (Multiple rounds) | 957.95 | As base case | 3,396.97 | Point estimate | Measured cost from study | Base case | Base case |
|  |  | Intervention delivery | 335.66 | As base case | 3,396.97 | Point estimate | Measured cost from study | Base case | Base case |
|  | Costs (Economic):Regular | Planning | 62.79 | As base case | 3,396.97 | Point estimate | Measured cost from study | Base case | Base case |
|  |  | Training | 6,841.59 | As base case | 3,396.97 | Point estimate | Measured cost from study | Base case | Base case |
|  |  | ZZApp training | 2,858.06 | As base case | 3,396.97 | Point estimate | Measured cost from study | Base case | Base case |
|  |  | Habitat characterisation | 1,779.79 | As base case | 3,396.97 | Point estimate | Measured cost from study | Base case | Base case |
|  |  | Mapping | 10,957.23 | As base case | 3,396.97 | Point estimate | Measured cost from study | Base case | Base case |
|  |  | Mapping man. and supervision | 9,012.91 | As base case | 3,396.97 | Point estimate | Measured cost from study | Base case | Base case |
|  |  | Intervention delivery | 2,325.49 | As base case | 3,396.97 | Point estimate | Measured cost from study | Base case | Base case |
|  |  | 6*rounds of Larviciding | 14,404.77 | 18,103.21 | 13,045.89 | Triangle (best, base, worst) | As above | As above | As above |
|  |  | Intervention supervision | 7,887.81 | As base case | 3,396.97 | Point estimate | Measured cost from study | Base case | Base case |
| Conv | Costs (Economic):Startup | Community sensitisation | - | - | - | n.a | Not needed when not using drones/app |  |  |
|  |  | Training | - | - | - | n.a | As above |  |  |
|  |  | Mapping (Multiple rounds) | - | - | - | n.a | As above |  |  |
|  |  | Intervention delivery | 355.12 | As base case | 3,396.97 | Point estimate | Measured cost from study | Base case | Base case |
|  | Costs (Economic):Regular | Planning | 108.26 | As base case | 3,396.97 | Point estimate | Measured cost from study | Base case | Base case |
|  |  | Training | 6,668.37 | As base case | 3,396.97 | Point estimate | Measured cost from study | Base case | Base case |
|  |  | ZZApp training | - | - | - | n.a | Not needed when not using drones/app |  |  |
|  |  | Habitat characterisation | 1,779.79 | As base case | 3,396.97 | Point estimate | Measured cost from study | Base case | Base case |
|  |  | Mapping | 13,721.71 | As base case | 3,396.97 | Point estimate | Measured cost from study | Base case | Base case |
|  |  | Mapping man. and supervision | 9,012.91 | As base case | 3,396.97 | Point estimate | Measured cost from study | Base case | Base case |
|  |  | Intervention delivery | 3,510.94 | As base case | 3,396.97 | Point estimate | Measured cost from study | Base case | Base case |
|  |  | 6*rounds of Larviciding | 22,086.27 | 32,006.94 | 18,021.85 | Triangle (best, base, worst) | Measured cost from study of 6 rounds in conv arm | Max conv round cost * 6 from study | Min conv round cost * 6 from study |
|  |  | Intervention supervision | 7,887.81 | As base case | 3,396.97 | Point estimate | Measured cost from study | Base case | Base case |
| Both | Population density (people/KM2) | Census data | 390.5 | 351.1 | 416.5 | Gamma fitted from rural Zanzibar population data (2022 census) | Mean of population density by arm including control arm & both intervention arms | Lowest population density by arm including control arm & both intervention arms | Highest population density by arm including control arm & both intervention arms |
|  | Intervention efficacy | Malaria cases averted per 1000 person per year (SIS versus Conventional) | -0.14 | -0.91 | 0.61 | Uniform | Study data: Point estimate of adjusted rate difference between the SIS arm compared to the conventional arm | Study data: Lower bound on point estimate of adjusted rate difference between the SIS arm compared to the conventional arm | Study data: Upper bound on point estimate of adjusted rate difference between the SIS arm compared to the conventional arm |
|  | DALY inputs | Malaria case fatality rate (All ages) | 0.0034 | 0.0048 | 0.0001 | Triangle (worst, base, best) | Calculated by authors using point estimates, malaria deaths/malaria cases. Data obtained from 2018 World Malaria Report (ref) | Calculated by authors using lower bound estimates, malaria deaths/malaria cases. Data obtained from 2018 World Malaria Report (ref) | Calculated by authors using upper bound estimates, malaria deaths/malaria cases. Data obtained from 2018 World Malaria Report (ref) |
|  |  | Proportion of cases that become severe | 0.06 | 0.05 | 0.07 | Uniform | Assumption | Assumption | Assumption |
|  |  | Duration of uncomplicated malaria morbidity (days) | 3.00 | 1.00 | 5.00 | Uniform | Assumption | Assumption | Assumption |
|  |  | Duration of severe malaria morbidity (days) | 7.00 | 4.00 | 12.00 | Uniform | Assumption | Assumption | Assumption |
|  |  | DALY weight uncomplicated malaria (infectious disease moderate) | 0.051 | 0.032 | 0.074 | Triangle (worst, base, best) | Point estimate DALY weight for Infectious disease, acute episode, moderate from 2015 Global Burden of Disease study (cite https://www.thelancet.com/journals/langlo/article/PIIS2214-109X(15)00069-8/fulltext) | Min of 95% uncertainty range, DALY weight for Infectious disease, acute episode, moderate from 2015 Global Burden of Disease study (cite https://www.thelancet.com/journals/langlo/article/PIIS2214-109X(15)00069-8/fulltext) | Max of 95% uncertainty range on DALY weight for Infectious disease, acute episode, moderate from 2015 Global Burden of Disease study (cite https://www.thelancet.com/journals/langlo/article/PIIS2214-109X(15)00069-8/fulltext) |
|  |  | DALY weight severe malaria (infectious disease moderate) | 0.133 | 0.088 | 0.190 | Triangle (worst, base, best) | Point estimate DALY weight for Infectious disease, acute episode, severe from 2015 Global Burden of Disease study (cite https://www.thelancet.com/journals/langlo/article/PIIS2214-109X(15)00069-8/fulltext) | Min of 95% uncertainty range, DALY weight for Infectious disease, acute episode, severe from 2015 Global Burden of Disease study (cite https://www.thelancet.com/journals/langlo/article/PIIS2214-109X(15)00069-8/fulltext) | Max of 95% uncertainty range, DALY weight for Infectious disease, acute episode, severe from 2015 Global Burden of Disease study (cite https://www.thelancet.com/journals/langlo/article/PIIS2214-109X(15)00069-8/fulltext) |
|  |  | Years of life lost per death (not discounted) | 67.33 | 35.58 | 68.83 | Triangle (worst, base, best) | Mean of expected additional years of life for age groups <1 to 9 years old, from WHO Global Health Observatory, indicator ex - expectation of life at age x, United Republic of Tanzania 2015. Available from https://www.who.int/data/gho/data/indicators/indicator-details/GHO/gho-ghe-life-tables-by-country, accessed 24/05/2023 | Mean of expected additional years of life for all age groups <1 to +85 old, from WHO Global Health Observatory, indicator ex - expectation of life at age x, United Republic of Tanzania 2015. Available from https://www.who.int/data/gho/data/indicators/indicator-details/GHO/gho-ghe-life-tables-by-country, accessed 24/05/2023 | Maximum of expected additional years of life for all age groups from <1 to +85 years old, from WHO Global Health Observatory, indicator ex - expectation of life at age x, United Republic of Tanzania 2015. Available from https://www.who.int/data/gho/data/indicators/indicator-details/GHO/gho-ghe-life-tables-by-country, accessed 24/05/2023 |

**Table 2 Intervention related costs only: Unannualised cost and annualised economic and financial costs by study arm (SIS or Conventional) cost type and activity (US$)**

| **Cost category** | **Activity** | **SIS** | | | **Conventional** | | |
| --- | --- | --- | --- | --- | --- | --- | --- |
|  |  | **Unannualised** | **Annualised** | | **Unannualised** | **Annualised** | |
|  |  |  | **Econ** | **Fin** |  | **Econ** | **Fin** |
| **Personnel** | Intervention delivery | 195 | 195 | 195 | 195 | 195 | 195 |
|  | IR 1 | 2,364 | 2,364 | 2,364 | 4,759 | 4,759 | 4,759 |
|  | IR 2 | 2,364 | 2,364 | 2,364 | 4,032 | 4,032 | 4,032 |
|  | IR 3 | 2,228 | 2,228 | 2,228 | 3,254 | 3,254 | 3,254 |
|  | IR 4 | 2,241 | 2,241 | 2,241 | 3,059 | 3,059 | 3,059 |
|  | IR 5 | 2,137 | 2,137 | 2,137 | 2,955 | 2,955 | 2,955 |
|  | IR 6 | 2,131 | 2,131 | 2,131 | 3,083 | 3,083 | 3,083 |
|  | Intervention supervision | 7,706 | 7,706 | 7,706 | 7,706 | 7,706 | 7,706 |
|  | **Sub-total** | **21,366** | **21,366** | **21,366** | **29,044** | **29,044** | **29,044** |
| **Consumables** | Intervention delivery | 1,736 | 1,736 | 1,736 | 1,736 | 1,736 | 1,736 |
|  | IR 1 | 95 | 95 | - | 18 | 18 | - |
|  | IR 2 | 14 | 14 | - | 81 | 81 | 65 |
|  | IR 3 | 12 | 12 | - | 15 | 15 | - |
|  | IR 4 | 12 | 12 | - | 16 | 16 | - |
|  | IR 5 | 11 | 11 | - | 16 | 16 | - |
|  | IR 6 | 11 | 11 | - | 14 | 14 | - |
|  | **Sub-total** | **1,892** | **1,892** | **1,736** | **1,896** | **1,896** | **1,801** |
| **Equipment** | Intervention delivery | **616** | **329** | **322** | **5,728** | **1,515** | **1,345** |
| **Transport** | IR 1 | 558 | 558 | 558 | 558 | 558 | 558 |
|  | IR 2 | 96 | 96 | 96 | 96 | 96 | 96 |
|  | IR 3 | 32 | 32 | 32 | 32 | 32 | 32 |
|  | IR 4 | 32 | 32 | 32 | 32 | 32 | 32 |
|  | IR 5 | 32 | 32 | 32 | 32 | 32 | 32 |
|  | IR 6 | 32 | 32 | 32 | 32 | 32 | 32 |
|  | Intervention supervision | 182 | 182 | 182 | 182 | 182 | 182 |
|  | **Sub-total** | **966** | **966** | **966** | **966** | **966** | **966** |
| **Other** | Intervention delivery | 1,602 | 401 | 372 | 1,602 | 420 | 372 |
| **Grand Total** | | **26,442** | **24,954** | **24,763** | **39,236** | **33,840** | **33,528** |
