# Supplementary material for: Cost and quality of operational larviciding using drones and smartphone technology
Source: Malar J. 2023 Sep 27;22:286. doi: 10.1186/s12936-023-04713-0 (PMC10523724; doi:10.1186/s12936-023-04713-0)
Supplement: Supplementary file 2 — Additional file 2: Table S1. Unannualized cost and annualized economic and financial costs by study arm (SIS or Conventional) phase (start up or regular) and activity (US$). Table S2. Summary of case incidence data and rate difference between the baseline year and the follow up period. [file 12936_2023_4713_MOESM2_ESM.docx]

**Table S1**. Unannualized cost and annualized economic and financial costs by study arm (SIS or Conventional) phase (start up or regular) and activity (US$).

| **Phase** | **Activity** | **SIS** | | | | | | **Conventional** | | | | | |
| --- | --- | --- | --- | --- | --- | --- | --- | --- | --- | --- | --- | --- | --- |
|  |  | **Unannualised total cost** | | **Annualised total cost** | | | | **Unannualised total cost** | | **Annualised total cost** | | | |
|  |  |  |  | **Economic** | | **Financial** | |  |  | **Economic** | | **Financial** | |
| **Startup** | Community sens.^§^ | 22,226 | 23.1% | 4,853 | 7.6% | 4,445 | 7.1% | - | 0.0% | - | 0.0% | - | 0.0% |
|  | Training^§^ | 6,516 | 6.8% | 1,423 | 2.2% | 1,303 | 2.1% | - | 0.0% | - | 0.0% | - | 0.0% |
|  | Mapping ^§^ | 4,387 | 4.6% | 958 | 1.5% | 877 | 1.4% | - | 0.0% | - | 0.0% | - | 0.0% |
|  | Intervention delivery | 1,537 | 1.6% | 336 | 0.5% | 307 | 0.5% | 1,537 | 2.2% | 355 | 0.5% | 307 | 0.5% |
|  | **Subtotal** | **34,667** | **36.0%** | **7,569** | **11.9%** | **6,933** | **11.1%** | **1,537** | **2.2%** | **355** | **0.5%** | **307** | **0.5%** |
| **Reg.** | Planning^§^ | 63 | 0.1% | 63 | 0.1% | 63 | 0.1% | 108 | 0.2% | 108 | 0.2% | 108 | 0.2% |
|  | Training^§^ | 6,842 | 7.1% | 6,842 | 10.7% | 6,582 | 10.5% | 6,668 | 9.5% | 6,668 | 10.2% | 6,582 | 10.2% |
|  | ZZApp training^§^ | 2,858 | 3.0% | 2,858 | 4.5% | 2,858 | 4.6% | - | 0.0% | - | 0.0% | - | 0.0% |
|  | Habitat charac.^§^ | 1,780 | 1.9% | 1,780 | 2.8% | 1,780 | 2.8% | 1,780 | 2.5% | 1,780 | 2.7% | 1,780 | 2.7% |
|  | Mapping^§^ | 16,037 | 16.7% | 10,957 | 17.2% | 10,838 | 17.3% | 13,722 | 19.5% | 13,722 | 21.1% | 13,722 | 21.2% |
|  | Mapping man & supervision^§^ | 9,013 | 9.4% | 9,013 | 14.1% | 9,013 | 14.4% | 9,013 | 12.8% | 9,013 | 13.8% | 9,013 | 13.9% |
|  | Intervention delivery | 2,613 | 2.7% | 2,325 | 3.7% | 2,319 | 3.7% | 7,724 | 11.0% | 3,511 | 5.4% | 3,341 | 5.2% |
|  | Intervention round 1 | 3,017 | 3.1% | 3,017 | 4.7% | 2,922 | 4.7% | 5,334 | 7.6% | 5,334 | 8.2% | 5,317 | 8.2% |
|  | Intervention round 2 | 2,475 | 2.6% | 2,475 | 3.9% | 2,460 | 3.9% | 4,208 | 6.0% | 4,208 | 6.5% | 4,192 | 6.5% |
|  | Intervention round 3 | 2,272 | 2.4% | 2,272 | 3.6% | 2,260 | 3.6% | 3,302 | 4.7% | 3,302 | 5.1% | 3,287 | 5.1% |
|  | Intervention round 4 | 2,285 | 2.4% | 2,285 | 3.6% | 2,273 | 3.6% | 3,108 | 4.4% | 3,108 | 4.8% | 3,092 | 4.8% |
|  | Intervention round 5 | 2,181 | 2.3% | 2,181 | 3.4% | 2,170 | 3.5% | 3,004 | 4.3% | 3,004 | 4.6% | 2,988 | 4.6% |
|  | Intervention round 6 | 2,174 | 2.3% | 2,174 | 3.4% | 2,163 | 3.5% | 3,130 | 4.4% | 3,130 | 4.8% | 3,116 | 4.8% |
|  | Intervention sup. | 7,888 | 8.2% | 7,888 | 12.4% | 7,888 | 12.6% | 7,888 | 11.2% | 7,888 | 12.1% | 7,888 | 12.2% |
|  | **Subtotal** | **61,497** | **64.0%** | **56,130** | **88.1%** | **55,589** | **88.9%** | **68,989** | **97.8%** | **64,776** | **99.5%** | **64,425** | **99.5%** |
| **Grand Total Cost** | | **96,164** | **100.0%** | **63,700** | **100.0%** | **62,522** | **100.0%** | **70,527** | **100.0%** | **65,131** | **100.0%** | **64,732** | **100.0%** |
| **Cost per M^2^ mapped** | | **799.5** | **n.a** | **444.3** | **n.a** | **433.0** | **n.a** | **371.3** | **n.a** | **371.3** | **n.a** | **370.3** | **n.a** |
| **Cost per person protected** | Unadjusted ^Δ^ | 2.72 | n.a. | 1.80 | n.a. | 1.77 | n.a. | 2.38 | n.a. | 2.20 | n.a. | 2.19 | n.a. |
|  | Adjusted ^∝^ | 2.96 | n.a. | 1.96 | n.a. | 1.92 | n.a. | 2.17 | n.a. | 2.00 | n.a. | 1.99 | n.a. |

^§^_­­_Mapping related activities, as opposed to intervention delivery related activities

^Δ^ Uses estimated number of people in each arm SIS = 35491 people and Conventional = 29597 people.

^∝^ Uses estimated mean number of people in each arm (SIS + Conventional)/2= 32508 people

**Table S2**. Summary of case incidence data and rate difference between the baseline year and the follow up period.

| **Arm** | **Population estimate** | **Number of cases in baseline year** | **Mean of cluster incidences (per 1000 person-years) (baseline)** | **Number of cases in follow up period** | **Mean of cluster incidences (per 1000 person-years) for follow up** | **Rate difference (compared to control)** | **Rate difference (compared to control), adjusted for baseline incidence** |
| --- | --- | --- | --- | --- | --- | --- | --- |
| SIS | 35,419 | 135 | 3.78 (1.66-5.90) | 45 | 1.28 (0.81-1.76) | 0.53 (-0.13-1.19), p=0.113 | 0.52 (-0.161-1.20), p=0.130 |
| Conventional | 29,597 | 155 | 5.61 (2.95-8.26) | 34 | 1.25 (0.62-1.89) | 0.50 (-0.27-1.27), p=0.200 | 0.22 (-0.54-0.97), p=0.563 |
| Control | 31,363 | 94 | 3.20 (2.16-4.24) | 23 | 0.76 (0.25-1.26)** | Reference | Reference |
| **Total** | **96,379** | **384** | **4.19 (3.06-5.32)** | **102** | **1.10 (0.80-1.39)** |  |  |

c ** 7 clusters in this arm had no cases during follow up
